# Proteome characterization of developing grains in bread wheat cultivars (*Triticum aestivum* L.)

**DOI:** 10.1186/1471-2229-12-147

**Published:** 2012-08-19

**Authors:** Guangfang Guo, Dongwen Lv, Xing Yan, Saminathan Subburaj, Pei Ge, Xiaohui Li, Yingkao Hu, Yueming Yan

**Affiliations:** 1 College of Life Science, Capital Normal University, Beijing 100048, China

**Keywords:** Wheat, Grain proteome, Phosphorproteins, 2-DE, Tandem MS, qRT-PCR

## Abstract

**Background:**

The analyses of protein synthesis, accumulation and regulation during grain development in wheat are more complex because of its larger genome size compared to model plants such as Arabidopsis and rice. In this study, grains from two wheat cultivars Jimai 20 and Zhoumai 16 with different gluten quality properties were harvested at five development stages, and were used to displayed variable expression patterns of grain proteins.

**Results:**

Proteome characterization during grain development in Chinese bread wheat cultivars Jimai 20 and Zhoumai 16 with different quality properties was investigated by 2-DE and tandem MALDI-TOF/TOF-MS. Identification of 117 differentially accumulated protein spots representing 82 unique proteins and five main expression patterns enabled a chronological description of wheat grain formation. Significant proteome expression differences between the two cultivars were found; these included 14 protein spots that accumulated in both cultivars but with different patterns and 27 cultivar-different spots. Among the cultivar-different protein spots, 14 accumulated in higher abundance in Jimai 20 than in Zhoumai 16, and included NAD-dependent isocitrate dehydrogenase, triticin precursor, LMW-s glutenin subunit and replication factor C-like protein. These proteins are likely to be associated with superior gluten quality. In addition, some proteins such as class II chitinase and peroxidase 1 with isoforms in developing grains were shown to be phosphorylated by Pro-Q Diamond staining and phosphorprotein site prediction. Phosphorylation could have important roles in wheat grain development. qRT-PCR analysis demonstrated that transcriptional and translational expression patterns of many genes were significantly different.

**Conclusions:**

Wheat grain proteins displayed variable expression patterns at different developmental stages and a considerable number of protein spots showed differential accumulation between two cultivars. Differences in seed storage proteins were considered to be related to different quality performance of the flour from these wheat cultivars. Some proteins with isoforms were phosphorylated, and this may reflect their importance in grain development. Our results provide new insights into proteome characterization during grain development in different wheat genotypes.

## Background

Wheat is one of the three most important crops in the world due to its value as a major food source and its unique suitability to bread production. The protein composition of wheat grain is the key to bread baking quality. Wheat grain proteins are classified into non-prolamins, including albumins and globulins, and prolamins including gliadins and glutenins [1,2]. Albumins and globulins are more abundant in essential amino acids such as lysine, tryptophan and methionine that are very important for human health. Prolamins are the major storage proteins and determine the viscoelasticity of dough. Understanding protein synthesis and regulation during grain development is of fundamental importance for targeting wheat quality by conventional breeding or genetic engineering to specific end-uses.

The development of wheat grain involves three distinct phases: cell division and differentiation, grain filling, and desiccation/maturation [3]. In recent years, studies on transcriptomics, proteomics and metabolomics have provided insights into the mechanism of reserve accumulation during wheat grain development. For instance, using Affymetrix wheat GeneChip oligonucleotide arrays, 14,550 transcripts showed significant differential regulation in developing caryopses of hexaploid wheat cv. Hereward between 6 and 42 days after anthesis [3]. Transcriptomic and metabolomic approaches have been used to investigate the impacts of nitrogen (N) and sulphur (S) deficiency on N and S remobilization during grain filling in winter wheat [4]. It has been noted, however, that the accumulated amounts of a large proportion of proteins are often poorly correlated with their corresponding mRNAs in expression profiles [5-7] and hence direct proteome approaches can be more valuable in monitoring developmental profiles. Two-dimensional electrophoresis (2-DE) and mass spectrometry (MS) proteomic approaches provide the tools for the monitoring the dynamic expression profiles of proteins during seed development, and have been widely used in different plant species such as *Arabidopsis*[5,8], *Medicago truncatula*[9], soybean [10], maize [11] and rice [12,13]. Considerable work on wheat grain proteomics has been carried out in different wheat varieties [14,15], endosperm and endosperm amyloplasts [16,17], kernel aleurone and peripheral layers [18], and grain storage proteins [19-21]. Recent work has focused on proteomic analysis of different grain developing stages, such as six grain developmental stages in winter wheat variety Récital [22], five developmental stages in Jing 411 and Sunstate at [23], four developmental stages in two near-isogenic lines of bread wheat cv. Falcon [24], four developmental stages in two spring wheat varieties Ningchun 4 and Chinese Spring under drought stress [25]. Although these studies provided valuable information, the biochemical processes important for wheat grain development still require research because this species has a much larger genome and a more complex proteome than model plants such as *Arabidopsis* and rice.

A complex gene network regulates protein expression during grain development [26]. Various post-translational modifications of proteins (PTMs) occur as grains develop and mature. The poor association between transcription (mRNA) and translation (protein) levels indicates the importance of PTMs. Protein phosphorylation, as an important PTM and a transient and reversible modification, plays a crucial role in signaling and regulation of cellular processes such as proliferation, differentiation, and apoptosis [27]. For example, phosphorylation in wheat amyloplasts is capable of regulating starch branching enzyme activity and protein–protein interactions [28]. Among the many strategies for studying protein phosphorylation, a powerful way for directly identifying phosphorylated proteins is to separate phosphoproteins by 2-DE and to stain with phosphospecific dyes such as Pro-Q Diamond phosphoprotein staining followed by tandem mass spectrometry analyses [29-31].

In the present work, we conducted an investigation on proteome characterization of developing grains in two bread wheat cultivars (Jimai 20 and Zhoumai 16) with different gluten quality properties, by 2-DE and MALDI-TOF/TOF-MS. Jimai 20 has high yield and superior gluten quality, whereas Zhoumai 16 has poor gluten quality and higher yield [32]. In the past ten years, both cultivars were widely cultivated in the main wheat areas of China. Our results provide a comprehensive view of proteome characterization during grain development in different wheat genotypes.

## Results

### Grain development and SEM observations

**Figure 1:**
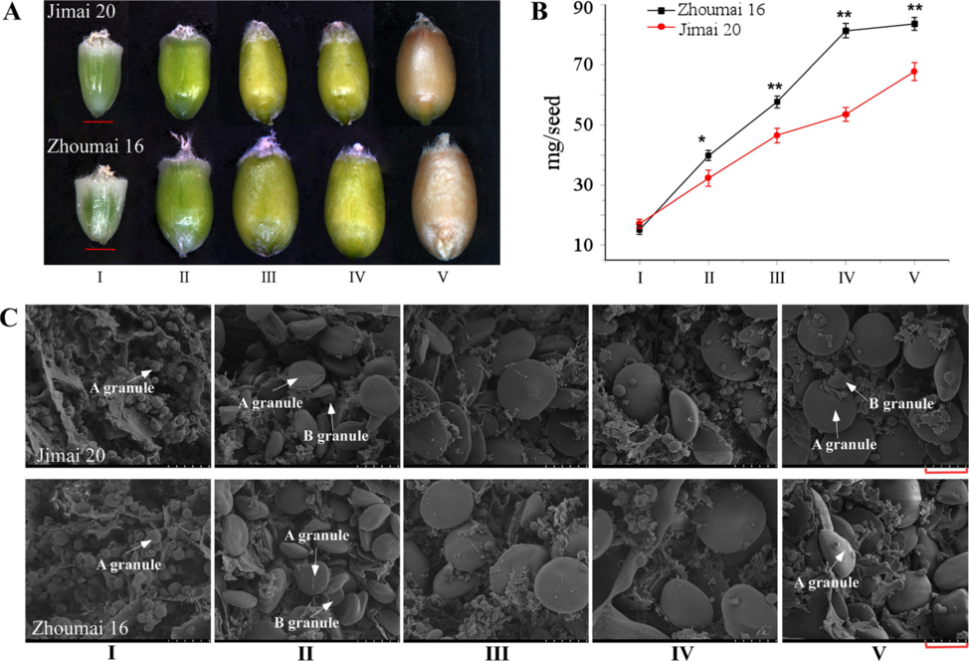
**Grain development during five stages (I, II, III, IV and V) in wheat cultivars Jimai 20 and Zhoumai 16. A**. Grain morphological development (the red lines represent 2 mm). **B**. Grain weight accumulation. **C**. SEM images of transverse grain sections at five developmental stages. Red lines represent 15 μm. A and B type starch granules are indicated.

In general, grain size and weight in both Jimai 20 and Zhoumai 16 increased gradually from flowering to maturity, but their development rates and grain sizes were different (Figure 1A, B). Zhoumai 16 had a larger grain size and higher grain weight than Jimai 20 at all grain developmental stages except the first. SEM observations on both cultivars indicated that starch granules accumulated continuously until grain maturity (Figure 1C). As previously observed [33,34], A (diameter >10 μm) and B (diameter <10 μm) starch granules appeared at 6 DPA (147^o^Cd) and 11 DPA (252^o^Cd), respectively. The size of A granules as well as grain weight increased rapidly from 11 to 15 DPA (252-353^o^Cd), but B granules grew only slowly from 11 to 31 DPA. This indicated that the period 11–15 DPA was a key stage for grain starch synthesis and accumulation.

### Identification, classification and localization of differentially accumulated proteins during grain development in the two cultivars

**Figure 2:**
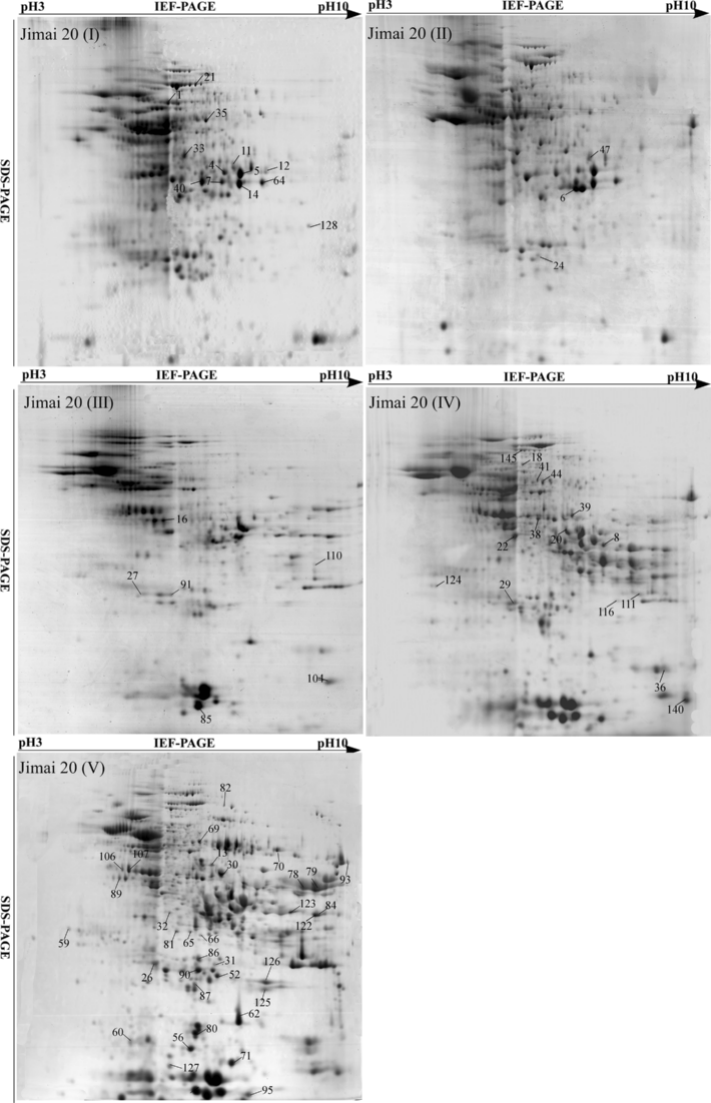
2-DE maps of proteins extracted from the first sample of Jimai 20.

**Figure 3:**
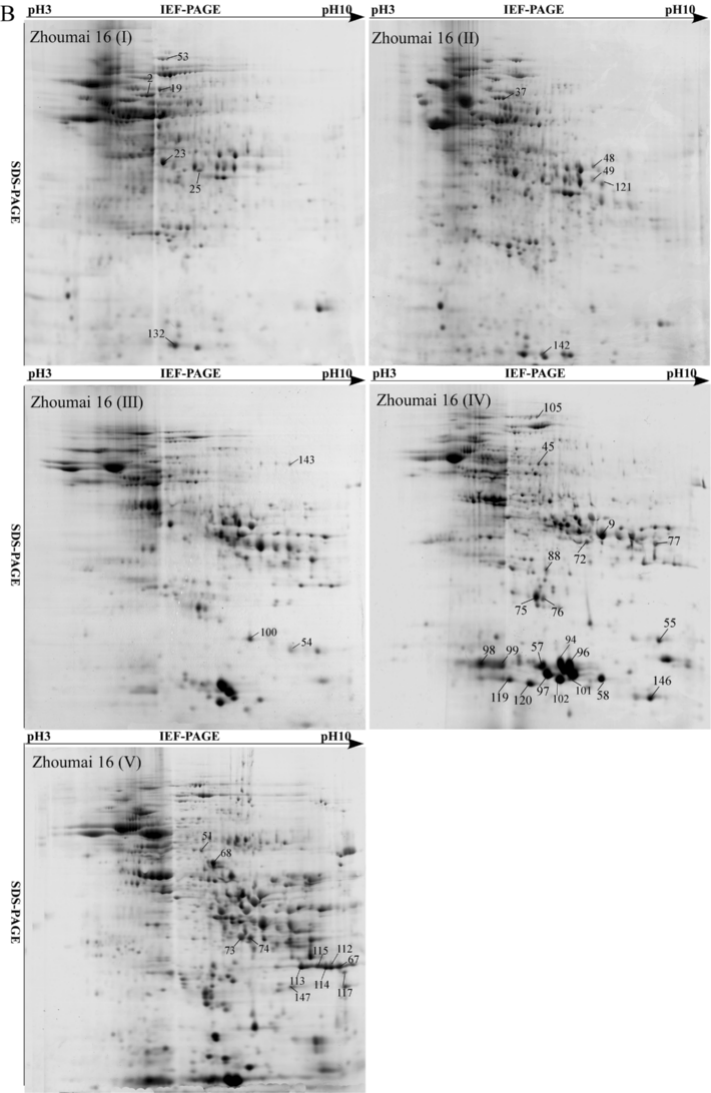
Zhoumai 16 at five stages of grain development.

Grain proteins extracted at the five developmental stages in the two cultivars were separated by 2-DE with broad-range (pH 3-10 L) IPG strips. The proteome profiles were generally similar in both cultivars at all five stages (Figure 2) (Figure 3). Most of the proteins on the 2-DE gels were distributed in the pH 3–7 range during the earlier (I and II) development stages. The numbers of basic proteins increased considerably from stage III, especially in the last stage. In total, 174 protein spots showed more than two fold differences in abundance, of which 117 representing 84 unique proteins were identified by MALDI-TOF/TOF-MS (Table 1). Their peptide sequences are listed in Additional file 1: Table S1.

**Table 1:** Differentially expressed proteins identified by MALDI-TOF/TOF-MS at five grain developmental stages in bread wheat cultivars Jimai 20 and Zhoumai 16

**Spot no.**^**a**^	**Accession no.**^**b**^	**Protein name**	**Species**	**Protein score C.I.%**^**c**^	**Total Ion score**	**MP**^**d**^	***EpI*/*TpI***^**e**^	**Average volume ratio (Jimai 20)**		**Average volume ratio (Zhoumai 16)**		**HCP (J/Z)**^**f**^	**Predicted subcellular Localization**^**g**^
								**I:II:III:IV:V**	***p*-value**	**I:II:III:IV:V**	***p*-value**		
**Carbohydrate metabolism**													
**1. TCA pathway:**													
22	gi/49343245	Cytosolic malate dehydrogenase	*T. aestivum*	100	161	6	5.36/5.75	1:1.1:2.4:1.3:0.3	0.015	1:1.2:0.8:0.3:0.2	0.036	C/C	Cyto
23	gi/49343245	Cytosolic malate dehydrogenase	*T. aestivum*	100	51	2	5.76/5.75	1:1.5:2.6:1.4:0.4	0.023	1:1.4:1.0:0.4:0.3	0.030	C/C	Cyto
24	gi/37928995	Cytosolic malate dehydrogenase	*T. aestivum*	100	266	3	5.98/6.62	1:0.7:.0.4:0.3:0	0.038	1:0.7:0.6:0:0	0.027	A/A	Cyto
25	gi/15232820	MDH (malate dehydrogenase)	*A. thaliana*	100	101	2	6.82/8.66	1:0.8:0.8:0.5:0.3	0.019	1:0.7:1.0:0.7:0.4	0.019	A/A	P
32	gi/7488742	Isocitrate dehydrogenase (NADP) precursor	*M. sativa*	99	144	3	5.54/6.15	1:0.6:0.7:0.5:0.1	0.041	1:0.9:0.5:0.4:0.2	0.011	A/A	P
33	gi/92875135	Isocitrate dehydrogenase NADP-dependent	*M. truncatula*	97.38	98	2	6.02/5.99	1:0.8:0.5:0.3:0.3	0.021	1:0.5:0.5:0.2:0.2	0.018	A/A	Mito
**2. Glycolysis:**													
1	gi/32400802	Phosphoglycerate mutase	*T. aestivum*	100	71	2	5.55/5.43	1:0.4:0.4:0.2:0.1	0.032	1:0.5:0.3:0.2:0	0.029	A/A	Cyto
2	gi/32400802	Phosphoglycerate mutase	*T. aestivum*	100	55	2	5.45/5.43	1:0.7:0.5:0.3:0.2	0.017	1:0.6:0.6:0.5:0.3	0.034	A/A	Cyto
4	gi/120680	Glyceraldehyde-3-phosphate dehydrogenase, cytosolic	*H. vulgare*	100	323	4	6.46/6.67	1:1.4:0.9:0.4:0.2	0.025	1:1.0:0.3:0.3:0.1	0.015	C/A	Cyto
5	gi/120680	Glyceraldehyde-3-phosphate dehydrogenase, cytosolic	*H. vulgare*	100	430	4	6.67/6.67	1:1.2:0.9:0.5:0.4	0.014	1:1.5:0.8:0.5:1.1	0.014	C/C	Cyto
6	gi/120668	Glyceraldehyde-3-phosphate dehydrogenase, cytosolic	*H. vulgare*	100	317	4	6.43/6.20	1:2.1:0.5:0.8:0.9	0.024	1:1.9:1.0:0.5:0.6	0.022	C/C	Cyto
7	gi/148508784	Glyceraldehyde-3-phosphate dehydrogenase	*T. aestivum*	100	99	4	6.44/7.08	1:1.6:0.6:0.5:0.2	0.026	1:1.2:0.4:0.4:0.1	0.011	C/C	Cyto
8	gi/148508784	Glyceraldehyde-3-phosphate dehydrogenase	*T. aestivum*	100	137	4	7.47/7.08	1:1.3:0.4:0.4:0.3	0.031	-	-	C/-	Cyto
9	gi/148508784	Glyceraldehyde-3-phosphate dehydrogenase	*T. aestivum*	100	126	2	7.36/7.08	1:1.4:3.2:5.8:0.2	0.037	1:1.3:6.2:3.2:2.4	0.030	C/C	Cyto
11	gi/18978	Glyceraldehyde 3-phosphate dehydrogenase	*H. vulgare*	99.83	351	4	6.82/6.67	1:1.3:1:0.6:0.2	0.011	1:0.9:1.7:1.1:0.1	0.010	C/C	Cyto
12	gi/18978	Glyceraldehyde 3-phosphate dehydrogenase	*H. vulgare*	100	131	1	7.23/6.67	1:0.8:0.5:0.2:0	0.029	1:0.5:0.2:0:0	0.024	A/A	Cyto
13	gi/7579064	Cytosolic glyceraldehyde-3- phosphate dehydrogenase GAPDH	*T. aestivum*	100	238	3	6.16/7.83	0:0:0:1:1.3	0.041	0:0:0:1.0:1.9	0.011	B/B	Cyto
14	gi/32478662	Cytosolic glyceraldehyde-3- phosphate dehydrogenase	*T. aestivum*	100	208	2	6.66/6.34	1:1.2:0.8:0.6:0.3	0.005	1:1.4:0.8:0.5:0.5	0.023	C/C	Cyto
16	gi/28172909	Cytosolic 3-phosphoglycerate kinase	*T. aestivum*	100	52	2	4.76/4.91	1:1.6:2.1:1.7:0.4	0.019	1:1.9:1.6:2.0:0.6	0.035	C/C	Cyto
18	gi/18076790	Phosphoglucomutase (PGM)	*T. aestivum*	100	73	2	5.84/5.66	1:1.3:2.3:0.9:0.8	0.017	1:1.7:3.9:0.6:0.4	0.029	C/C	Cyto
19	gi/18076790	Phosphoglucomutase (PGM)	*T. aestivum*	98.67	47	1	5.85/5.66	1:0.7:0.2:0:0	0.026	1:0.5:0.2:0:0	0.015	A/A	Cyto
20	gi/226316441	Fructose-bisphosphate aldolase	*T. aestivum*	100	64	2	6.33/6.85	0:0:1.0:3.1:3.0	0.023	0:0:1.0:4.1:4.7	0.016	B/B	Cyto
21	gi/18496065	Putative fructose 1-,6-biphosphate aldolase	*T. aestivum*	98.12	84	1	6.15/8.71	1:0.5:0.2:0:0	0.034	1:0.4:0.2:0:0	0.034	A/A	Cyto
26	gi/1785948	Cytosolic triosephosphate isomerase	*H. vulgare*	100	148	2	5.18/5.39	1:1.1:0.7:0.5:1.4	0.036	1:2.1:1.6:2.0:1.7	0.025	E/E	Cyto
27	gi/11124572	Triosephosphat-isomerase	*T. aestivum*	100	116	2	4.46/5.38	1:1.4:0.6:0:0	0.013	1:0.8:0.4:0:0	0.022	C/A	Cyto
29	gi/11124572	Triosephosphat-isomerase	*T. aestivum*	100	235	3	5.72/5.38	1:1.4:0.8:0.5:0.2	0.012	1:0.9:0.5:0.3:0	0.037	C/A	Cyto
37	gi/133872550	Bp2A protein	*T. aestivum*	100	132	3	5.38/5.86	1:1.5:1:0.7:0.2	0.022	1:0.8:0.6:0.5:0	0.033	C/A	Cyto
**3. Alcoholic fermentation:**													
38	gi/32400847	Formate dehydrogenase	*T. aestivum*	100	68	2	6.02/8.61	0:0:1.0:2.6:2.9	0.033	0:0:0:1.0:1.5	0.019	B/B	Cyto
39	gi/32400847	Formate dehydrogenase	*T. aestivum*	100	112	3	6.45/8.61	0:0:1.0:3.4:6.1	0.031	0:0:1.0:3.2:7.8	0.028	B/B	Cyto
**4. Starch metabolism:**													
35	gi/21680	ADP-glucose pyrophosophorylase preprotein	*T. aestivum*	95.78	54	2	6.35/6.61	1:0.8:0.7:0.6:0.4	0.027	1:0.8:0.6:0.6:0.3	0.027	A/A	P
41	Q0PG36	Glucose-1-phosphate adenylyltransferase	*T. aestivum*	100	183	5	6.02/6.12	1:1.3:0.4:0.2:0.2	0.029	1:1.2:0.4:0.2:0.1	0.034	C/C	P
44	Q0PG36	Glucose-1-phosphate adenylyltransferase	*T. aestivum*	100	110	3	6.42/6.12	1:0.9:0.6:0.4:0.2	0.021	1:1.5:0.8:0.4:0.3	0.024	A/C	P
45	Q0PG36	Glucose-1-phosphate adenylyltransferase	*T. aestivum*	100	125	3	6.23/5.89	1:0.8:0.5:0.2:0.1	0.037	1:1.3:0.6:0.3:0.2	0.016	A/C	P
**5. Lipid and sterol metabolism:**													
30	gi/167113	Aldose reductase-related protein	*B. inermis*	98.12	101	1	6.19/6.28	0:0:0:1.0:1.8	0.034	0:0:0:1.0:6.8	0.025	B/B	Cyto
31	gi/167113	Aldose reductase-related protein	*B. inermis*	99.83	116	2	6.17/6.28	0:0:0:1.0:0.8	0.036	0:0:1.0:1.8:1.5	0.028	C/C	Cyto
40	gi/218157	Cytoplasmic aldolase	*O. sativajaponica*	100	110	2	6.65/6.56	1:0.6:0.3:0.2:0.1	0.031	1:0.9:0.4:0.3:0.1	0.019	A/A	Cyto
145	gi/66840998	5a2 protein	*T. aestivum*	100	187	4	5.84/8.38	1:8.3:3.5:2.1:2.4	0.038	1:1.1:0.5:0.7:0.7	0.024	C/C	P
**Protein synthesis/assembly/degradation**													
36	gi/41052632	Peptidylprolyl isomerase Cyp2	*O. sativajaponica*	96.38	50	2	8.97/8.61	1:0.2:0.8:0.9:1.1	0.012	1:0.5:0.5:0.4:0.8	0.014	D/D	Cyto
54	gi/154761388	Cyclophilin	*T. aestivum*	98.21	19	2	8.39/8.59	1:0.3:1.3:1.4:1.6	0.027	1:0.5:0.4:0.4:0.8	0.011	D/D	Cyto
55	gi/154761388	Cyclophilin	*T. aestivum*	100	84	3	9.12/8.59	1:0.2:0.8:0.9:1.1	0.031	1:0.6:0.5:0.5:0.9	0.009	D/D	Cyto
56	gi/154761388	Cyclophilin	*T. aestivum*	100	95	3	6.28/8.59	0:0:0:1.0:6.8	0.013	-	-	B/-	Cyto
59	gi/32352154	Nascent polypeptide associated complex alpha chain	*O. sativajaponica*	100	83	1	4.17/4.34	0:0:0:1.0:2.1	0.016	0:0:0:1.0:3.0	0.028	B/B	Cyto
62	gi/2492077	Sequence 5 from patent US 5668007	*T. aestivum*	100	189	2	6.68/6.77	0:1.0:3.5:2.1:5.3	0.027	0:1.0:0.9:3.0:6.7	0.017	B/B	Cyto
106	gi/75279909	Serpin-Z2B; AltName: Full = TriaeZ2b;	*T. aestivum*	100	213	4	5.24/5.18	0:0:0:1.0:0.6	0.021	0:0:1.0:1.7:0.5	0.016	C/C	P
107	gi/75279909	Serpin-Z2B; AltName: Full = TriaeZ2b;	*T. aestivum*	100	256	4	5.32/5.18	1:1.8:2.4:1.4:0.9	0.023	0:0:1.0:1.7:1.4	0.021	C/C	P
128	gi/12229936	Proteasome subunit alpha type 7 (20S proteasome alpha subunit D)	*C. arietinum*	100	110	2	12.10/6.86	1:1.1:5.1:5.9:8.3	0.032	1:1.3:3.2:3.9:6.3	0.033	B/B	P
**Storage proteins**													
67	gi/171027826	Triticin	*T. aestivum*	100	84	2	8.94/6.43	0:0:1.0:1.4:3.0	0.042	0:0:1.0:2.0:3.3	0.045	B/B	ER
68	gi/171027826	Triticin	*T. aestivum*	100	475	6	6.35/6.43	0:0:1.0:2.1:5.5	0.039	0:0:1.0:2.4:6.5	0.041	B/B	ER
69	gi/7548844	Triticin precursor	*T. aestivum*	99.92	169	3	6.05/9.37	1:0.8:0.5:0.6:0.4	0.026	1:0.4:0.5:0.4:0.3	0.038	A/A	ER
70	gi/215398470	Globulin 3	*T. aestivum*	100	471	5	7.24/7.78	0:0:0:1.0:2.6	0.019	0:0:0:1.0:2.0	0.008	B/B	ER
71	gi/215398470	Globulin 3	*T. aestivum*	100	148	5	7.43/7.78	0:0:0:1.0:6.1	0.025	0:0:0:1.0:3.9	0.010	B/B	ER
72	gi/89143122	Putative avenin-like b precursor	*T. aestivum*	100	166	3	6.88/8.08	0:0:1.0:3.3:2.1	0.010	1.0:1.7:6.5:2.9:5.	0.034	C/C	ER
73	gi/145321072	Avenin-like protein	*T. aestivum*	100	97	2	6.63/8.29	0:0:1.0:3.2:4.1	0.024	0:0:1.0:3.5:4.0	0.031	B/B	ER
74	gi/209971847	Gamma-gliadin	*T. aestivum*	99.92	70	1	6.84/7.55	0:0:1.0:2.0:4.8	0.029	1.0:1.7:6.5:9.4:12.8	0.028	B/B	ER
75	gi/133741924	Gamma gliadin	*T. aestivum*	100	93	2	5.33/8.88	1:2.5:4.2:9.9:11.9	0.020	1:2.2:3.9:5.9:6.4	0.022	B/B	ER
76	gi/133741924	Gamma gliadin	*T. aestivum*	98.12	52	2	5.56/8.88	0:1:2.9:3.8:4.7	0.034	0:0:1.0:1.4:2.2	0.036	B/B	ER
77	gi/209971907	Gamma-gliadin	*T. aestivum*	97.35	55	2	8.55/8.64	0:1:2.0:2.2:4.2	0.031	0:0:1.0:1.6:2.1	0.031	B/B	ER
78	gi/164470668	LMW-s glutenin subunit 0359D24-S	*T. aestivum*	99.83	60	2	8.40/8.48	0:0:1:3.6:5.0	0.027	0:0:0:1.0:1.2	0.030	B/B	ER
79	gi/47607146	S-type low molecular weight glutenin L4-55	*T. aestivum*	100	90	2	8.67/8.51	0:0:0:1.0:2.9	0.024	-	-	B/-	Cyto
80	gi/215398468	Globulin 3C	*T. aestivum*	100	80	2	6.37/9.15	0:0:0:1.0:1.6	0.026	0:0:0:1.0:2.0	0.041	B/B	ER
81	gi/421978	Globulin Beg1 precursor	*H. vulgare*	100	85	2	5.64/6.81	0:0:0:1.0:4.2	0.031	0:0:0:1.0:3.0	0.044	B/B	ER
82	gi/167004	Embryo globulin	*H. vulgare subsp. vulgare*	95.67	53	1	6.79/6.81	0:0:0:1.0:4.4	0.011	0:0:0:1.0:2.5	0.025	B/B	ER
84	gi/170696	Storage protein	*T. aestivum*	100	52	1	9.49/6.82	0:0:1.0:1.4:1.7	0.012	0:0:1.0:0.8:2.0	0.018	B/B	ER
**Nitrogen metabolism**													
47	gi/164471780	Aspartate aminotransferase	*T. aestivum*	99.96	25	2	6.61/6.77	1:1.3:1.0:0.6:0.1	0.019	1:0.9:0.8:0.6:0.2	0.027	C/A	Cyto
48	gi/164471780	Aspartate aminotransferase	*T. aestivum*	100	25	2	6.95/6.77	1:0.8:0.5:0.2:0.3	0.021	1:0.6:0.5:0.2:0.2	0.024	A/A	Cyto
49	gi/584706	Aspartate aminotransferase, cytoplasmic	*O. sativajaponica*	100	159	3	6.89/7.75	-	-	1:0.7:0.5:0:0	0.031	-/A	Cyto
51	gi/14018051	Putative alanine aminotransferase	*O. sativajaponica*	100	107	3	6.11/6.23	1:2.4:3.3:2.6:1.5	0.034	1:3.4:2.1:2.1:1.1	0.034	C/C	Cyto
52	gi/56315117	Predicted serine-pyruvate aminotransferase	*O. sativajaponica*	100	113	1	6.18/5.86	0:0:0:1.0:2.7	0.031	0:0:0:1.0:1.7	0.018	B/B	Cyto
53	gi/6006863	Putative methionine synthase	*A. thaliana*	98.76	69	1	5.88/6.09	1:2.4:3.3:2.6:1.5	0.030	1:0.7:0.5:0:0	0.016	C/A	Cyto
**Transcription/translation**													
60	gi/479830	Translation elongation factor eEF-1 beta' chain	*T. aestivum*	100	64	1	4.63/4.54	0:0:0:1.0:2.2	0.016	0:0:0:1.0:1.7	0.028	B/B	Cyto
85	gi/18419557	Transposase	*T. aestivum*	97.54	98	4	6.53/9.48	1:1.8:6.1:1.6:0.2	0.015	1:1.5:2.2:1.7:1.1	0.027	C/C	Nucl
86	gi/30793446	27 K protein	*T. aestivum*	98.12	71	1	6.01/6.06	-	-	1:1.9:2.6:2.7:3.3	0.035	-/B	Cyto
87	gi/30793446	27 K protein	*T. aestivum*	95.89	69	1	5.98/6.06	0:0:0:1.0:2.1	0.045	0:0:0:1.0:2.3	0.031	B/B	Cyto
88	gi/30793446	27 K protein	*T. aestivum*	100	171	4	5.64/6.06	1:1.9:2.0:3.6:3.4	0.025	1:1.1:2.1:1.6:2.0	0.012	B/E	Cyto
89	gi/46394372	TPA: TPA_inf: WRKY transcription factor 59	*O. sativa*	95.45	113	2	5.46/7.7	1:1.4:2.2:3.0:2.9	0.018	1:1.8:2.2:4.4:2.7	0.028	B/C	P
90	gi/30793446	27 K protein	*T. aestivum*	100	60	1	6.06/6.06	0:0:0:1.0:2.3	0.021	0:0:0:1.0:2.2	0.015	B/B	Cyto
91	gi/27735373	Replication factor C like protein	*T. aestivum*	100	64	1	5.84/8.92	1:0.5:0.3:0.2:0.2	0.026	1:1.4:0.8:0.7:0.5	0.024	A/C	Nucl
93	Q03033	Elongation factor 1-alpha	*T. aestivum*	100	97	2	9.35/9.20	0:0:0:1.0:2.1	0.034	0:0:0:1.0:2.6	0.026	B/B	Nucl
**Signal transduction**													
64	gi/134290443	Pm3b-like disease r esistance protein 15Q1	*T. aestivum*	96.58	96	2	7.21/6.27	1:1.3:0.40.4:0.3	0.029	1:1.0:0.4:0.3:0.4	0.024	C/A	Cyto
65	gi/18145	Putative protein has homology to G protein beta subunit	*C. reinhardtii*	100	60	1	5.89/7.59	0:1.0:3.2:1.6:0	0.042	1:1.0:1.6:1.2:0	0.026	C/C	Cyto
66	gi/50932677	Putative guanine nucleotide- binding protein beta subunit	*O. sativa*	100	121	2	6.07/6.06	0:1.0:2.7:1.4:0	0.044	1:1.1:3.4:2.2:0.8	0.034	C/C	Cyto
142	gi/9652119	Nucleoside diphosphate kinase	*L. perenne*	99.92	192	2	6.36/6.30	1:0.9:0.7:0.4:0.2	0.019	1:0.8:0.8:0.5:0.2	0.021	A/A	Cyto
**Stress/defense**													
57	gi/640015	CMx	*T. aestivum*	100	102	2	5.51/9.23	0:0:1.0:2.5:1.8	0.036	0:0:1.0:2.8:2.4	0.022	C/C	P
58	gi/75107149	RecName: Full = Chymotrypsin inhibitor WCI; AltName: Full = Chloroform/ methanol-soluble protein WCI	*T. aestivum*	100	164	3	7.34/7.42	0:0:1.0:3.3:1.9	0.027	0:0:1.0:2.7:0.9	0.015	C/C	Cyto
94	gi/38098487	Alpha amylase inhibitor protein	*T. aestivum*	99.91	62	2	6.65/7.44	0:0:1.0:3.4:4.0	0.021	0:0:1.0:3.0:3.5	0.018	B/B	P
95	gi/66841026	Alpha-amylase inhibitor 0.19	*T. aestivum*	100	94	2	7.72/6.86	0:0:1.0:2.0:1.2	0.031	0:0:1.0:2.7:2.0	0.024	C/C	P
96	gi/66841026	Alpha-amylase inhibitor 0.19	*T. aestivum*	100	493	5	6.98/6.86	0:0:1.0:4.3:5.0	0.021	0:1.0:1.7:3.9:2.7	0.038	B/C	P
97	gi/123956	Alpha-amylase/trypsin inhibitor CM2;	*T. aestivum*	100	191	2	5.71/6.86	0:0:1.0:2.2:1.2	0.018	0:1.0:1.4:3.5:1.1	0.016	C/C	Extr
98	gi/221855644	Alpha-amylase inhibitor CM16 subunit	*T. macha*	99.96	60	2	4.56/5.31	0:0:1.0:4.2:1.4	0.021	0:0:1.0:2.3:1.7	0.035	C/C	Extr
99	gi/221855644	Alpha-amylase inhibitor CM16 subunit	*T. macha*	100	97	2	5.11/5.31	0:0:1.0:3.1:1.6	0.024	0:1.0:2.5:5.4:3.2	0.026	C/C	Extr
100	gi/225042	Alpha amylase inhibitor	*T. aestivum*	100	64	2	6.65/6.77	1:2.7:9.4:5.5:13.9	0.029	1:4.1:10.4:6.1:13.1	0.036	E/E	Cyto
101	gi/123955	Alpha-amylase/trypsin i nhibitor CM1;	*T. aestivum*	100	73	2	6.81/7.49	0:0:1.0:3.1:3.0	0.012	1:1.0:1.4:4.1:3.3	0.012	B/C	Extr
102	gi/54778507	0.19 dimeric alpha-amylase inhibitor	*T. aestivum*	100	238	2	6.76/5.73	1:1.5:2.6:2.4:2.3	0.025	1:1.6:2.1:2.7:2.4	0.028	C/C	P
104	gi/408873	Puroindoline = basic cystine-rich protein	*T. aestivum*	100	125	4	9.05/5.60	1:1.5:2.8:2.9:2.6	0.016	1:1.2:1.7:4.0:1.4	0.023	C/C	P
105	gi/408873	Puroindoline = basic cystine-rich protein	*T. aestivum*	100	125	4	6.73/8.76	0:0:1.0:3.0:5.2	0.039	0:0:1.0:2.0:2.5	0.045	B/B	P
110	gi/51247633	Chain A, Crystal Structure Of Family 11 Xylanase In Complex With Inhibitor (Xip-I)	*T. aestivum*	100	78	2	8.24/5.18	0:0:1.0:0.9:3.1	0.038	0:0:1.0:3.2:7.2	0.008	B/B	P
111	gi/51247633	Chain A, Crystal Structure Of Family 11 Xylanase In Complex With Inhibitor (Xip-I)	*T. aestivum*	100	163	3	8.32/5.18	0:0:1.0:1.4:1.5	0.027	0:0:1.0:4.5:5.8	0.015	B/B	P
112	gi/62465514	Class II chitinase	*T. aestivum*	100	95	2	8.39/5.56	0:0:1.0:1.1:1.8	0.025	0:0:1.0:0.7:1.7	0.028	B/B	Extr
113	gi/62465514	Class II chitinase	*T. aestivum*	100	54	1	8.42/8.27	0:0:1.0:1.1:1.6	0.024	0:0:1.0:1.2:2.0	0.015	B/B	Extr
114	gi/62465514	Class II chitinase	*T. aestivum*	100	138	2	8.37/8.27	1:2.5:5.8:3.0:9.8	0.025	1:1.2:2.1:2.1:6.2	0.025	B/B	Extr
115	gi/62465514	Class II chitinase	*T. aestivum*	100	69	3	8.35/8.27	0:0:1.0:1.2:1.9	0.038	0:0:1.0:0.8:3.9	0.005	B/B	Extr
116	gi/62465514	Class II chitinase	*T. aestivum*	100	206	4	7.40/8.66	0:0:1.0:1.4:1.5	0.027	0:0:1.0:1.6:2.1	0.034	B/B	Extr
117	gi/25989705	LEA1 protein	*T. aestivum*	100	117	2	9.21/8.81	0:0:0:1.0:2.9	0.012	0:0:0:1.0:2.7	0.021	B/B	Nucl
119	gi/134034615	Monomeric alpha-amylase inhibitor	*T. aestivum*	99.98	45	2	5.54/5.37	0:0:0:1.0:2.0	0.019	0:0:0:1.0:1.6	0.025	B/B	P
120	gi/134034615	Monomeric alpha-amylase inhibitor	*T. aestivum*	100	47	2	5.65/5.37	-	-	0:1.0:1.6:2.1:2.7	0.015	-/B	P
121	gi/22001285	Peroxidase 1	*T. aestivum*	99.99	58	3	7.35/8.14	1:1.3:0.4:0.4:0.3	0.038	1:1.0:0.4:0.1:0.4	0.045	C/A	Extr
122	gi/22001285	Peroxidase 1	*T. aestivum*	100	266	4	8.39/8.14	0:1.0:3.0:3.9:3.9	0.031	0:0:1.0:0.6:1.3	0.004	B/B	Extr
123	gi/22001285	Peroxidase 1	*T. aestivum*	100	136	6	8.42/8.14	0:0:1.0:0.6:0.8	0.029	0:0:1.0:0.8:1.6	0.026	C/B	Extr
124	gi/75246527	Translationally-controlled tumor protein	*T. aestivum*	100	203	3	4.24/4.55	1:1.5:0.7:0.3:0.2	0.025	1:2.2:1.4:1.1:0.6	0.049	C/C	Cyto
125	gi/20257409	Thaumatin-like protein	*T. aestivum*	100	255	6	7.65/7.85	0:0:0:1.0:6.7	0.024	0:0:1.0:2.3:2.0	0.030	B/B	P
126	gi/20257409	Thaumatin-like protein	*T. aestivum*	100	162	2	7.80/7.85	0:0:0:1.0:8.9	0.016	0:0:1.0:1.3:0.9	0.034	B/C	P
127	gi/226897529	Superoxide dismutase	*T. aestivum*	100	72	2	5.97/5.71	-	-	1.0:2.1:1.9:4.2:3.2	0.015	-/C	Cyto
**Photosynthesis**													
132	gi/11990897	Ribulose-1,5-bisphosphate carboxylase /oxygenase small subunit	*T. aestivum*	100	209	7	6.20/8.80	1.0:1.2:0.4:0.4:0.3	0.049	1:1.1:0.7:0.6:0.2	0.034	A/C	P
140	gi/34393258	Putative Oxygen-evolving enhancer protein 3–1, chloroplast precursor	*O. sativajaponica*	99.83	59	1	9.45/9.82	1:0.6:0.5:0.4:0.4	0.018	1:0.7:0.6:0.4:0.4	0.031	A/A	P
**Others**													
143	gi/357152329	Probable beta-D-xylosidase 7-like	*T. aestivum*	100	47	2	7.89/7.53	0:0:1.0:0.5:0.5	0.042	0:0:1.0:0.5:0.5	0.049	C/C	P
146	gi/2454602	Barperm1	*H. vulgare subsp. vulgare*	100	207	4	8.09/8.15	0:0:1.0:1.6:0.4	0.018	0:0:1.0:1.6:0.5	0.045	C/C	P
147	gi/115458852	Os04g0465600	*O. sativajaponica*	100	92	1	7.41/4.75	0:0:1.0:1.1:1.8	0.021	0:0:1.0:1.1:1.8	0.025	B/B	Cyto

^a^Spot no: corresponds to protein spot on gels shown in Figure 2 and Figure 3.

^b^Accession no: predicted protein in NCBInr and UniprotKB database.

^c^Protein Score C.I.%: the PMF score percentage of protein sequence (Confidence interval: Protein Score C.I.% ≥ 95%).

^d^MP: Matched peptides.

^e^*EpI*/*TpI*: p*I* of protein on the gel/p*I* of predicted protein.

^f^HCP(J/Z): hierachical cluster pattern A-E (Jimai 20/Zhoumai 16).

^g^Cyto: cytosol; P: plastid; Mito: mitochondria; ER: endoplasmic reticulum; Nucl: nuclear; Extr: extracellular.

**Figure 4:**
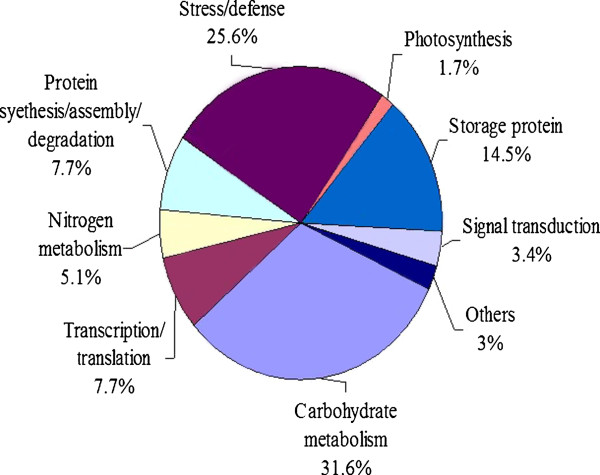
**Distribution of the proteins identified during five grain development stages in Jimai 20 and Zhoumai 16.** Nine protein groups were categorized based on putative functions.

According to their functions, the identified proteins were classified into several main groups, including carbohydrate metabolism (31.6%), stress/defense (25.6%), storage proteins (14.5%), protein synthesis/assembly/degradation (7.7%), transcription/translation (7.7%), nitrogen metabolism (5.1%) and signal transduction (3.4%) as shown as in Figure 4. More than 80% were identified as enzymes. Proteins related to carbohydrate metabolism contained five subcategories: glycolysis (18%), TCA pathway (5%), lipid and sterol metabolism (3%), starch metabolism (3%) and alcoholic fermentation (2%).

**Figure 5:**
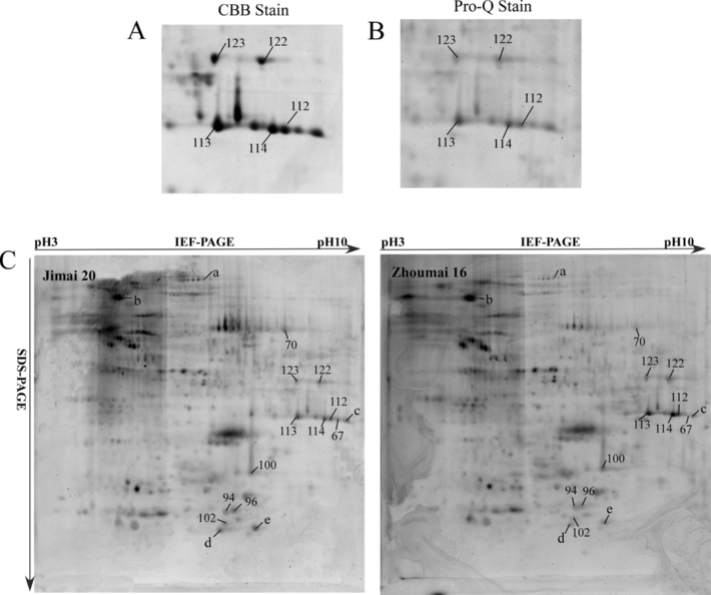
**Protein isoforms and phosphoproteins identified by 2-DE and Pro-Q diamond staining.** A total of 16 protein spots were identified as phosphoproteins, in which 5 spots (a-e) were newly identified phosphorylated proteins. Spot numbers correspond to those shown in Figure 2 and Figure 3 and Table 1.**A**: Spots from phase V of Jimai 20 stained with Coomassie Brilliant Blue (CBB stain). **B**: Spots from phase V of Jimai 20 stained with Pro-Q diamond phosphoprotein stain (Pro-Q stain). **C**: Gel images of proteins stained with Pro-Q Diamond at stage V by DryStrip (pH 3-10 L). Spots 122 and 123: Peroxidase 1 (gi/22001285); spots 112, 113 and 114: Class II chitinase (gi/62465514).

Among the differentially accumulated proteins, 25 different kinds were represented by two or more spots, and more than 10 isoforms were identified with different molecular masses or isoelectric points, each having two or three protein spots located at different positions on the same gel (Figure 2 and 5A), such as phosphoglycerate mutase (spots 1 and 2), glucose-1-phosphate adenylyltransferase (spots 41 and 44), triticin (spots 67 and 68), alpha-amylase inhibitor CM16 subunit (spots 98 and 99), monomeric alpha-amylase inhibitor (spots 119 and 120), class II chitinase (spots 112–116) and peroxidase 1 (spots 121, 122 and 123). Some of these isoforms might have resulted from certain PTMs such as phosphorylation.

In order to confirm the presence of phosphorylated proteins with isoforms, grain proteins at stage V were separated by 2-DE and then subjected to Pro-Q Diamond staining (Invitrogen) to detect putative phosphorylated proteins. Many spots stained in different intensities on the gels, indicating they were phosphorylated (Figure 5B). Sixteen spots with intense signals in both cultivars were identified by MALDI-TOF/TOF-MS (Table 2); their peptide sequences are listed in Additional file 2: Table S2. These phosphoproteins were mainly involved in stress and defense and the isoforms were particularly well stained, such as class II chitinase (gi/62465514) at spots 112, 113 and 114, and peroxidase 1 (gi/22001285) at spots 122 and 123 (Figure 5A).

**Table 2:** Phosphorylated proteins stained by Pro-Q Diamond and identified by MALDI-TOF/TOF-MS during grain filling

**Spot no.**	**Accession no.**	**Protein name**	**Protein score** **C.I.%**	**Total Ion score**	**Total Ion C.I.%**	**Theoretical *p*I/MW(kDa)**	**Experimental *p*I/MW(kDa)**	**Matched sequences**	**Predicted number of phosphorylation sites**^**a**^		
									**Ser**	**Thr**	**Tyr**
67^*^	gi/171027826	Triticin	100	84	100	6.43/64.90	8.79/30.52	2	18	2	4
70^*^	gi/215398470	Globulin 3	100	471	100	7.78/66.31	7.58/63.57	6	17	6	2
94^*^	gi/38098487	Alpha amylase inhibitor protein	100	152	100	7.44/18.21	6.10/16.70	3	2	1	0
96^*^	gi/54778501	0.19 dimeric alpha-amylase inhibitor	100	360	100	6.66/13.33	6.29/16.79	4			
100^*^	gi/225042	Alpha amylase inhibitor	100	259	100	6.77/19.62	6.87/20.32	4	8	2	3
102^*^	gi/54778507	0.19 dimeric alpha-amylase inhibitor	100	373	100	5.73/13.24	6.07/15.90	4	2	0	2
112^*^	gi/62465514	Class II chitinase	100	95	100	8.66/28.20	8.81/30.60	2	9	3	1
113^*^	gi/62465514	Class II chitinase	100	69	99.38	8.66/28.20	9.23/30.23	1	9	3	1
114^*^	gi/62465514	Class II chitinase	100	138	100	8.66/28.20	8.52/30.56	2	9	3	1
122^*^	gi/22001285	Peroxidase 1	100	138	100	8.14/38.80	8.55/51.53	4	11	5	2
123^*^	gi/22001285	Peroxidase 1	100	206	100	8.14/38.80	8.22/51.66	4	11	5	2
a^#^	gi/4558484	Heat shock protein 101	100	288	100	5.95/101.06	5.20/90.65	6	21	3	7
b^#^	gi/4204859	Heat shock protein 80	100	286	100	5.00/80.33	3.29/83.11	5	24	9	6
c^#^	gi/18146829	Chitinase 3	100	63	100	6.89/33.46	8.42/31.12	1	10	6	4
d^#^	gi/34925030	RecName: Full = Wheatwin-1; AltName:Full = Pathogenesis-related protein 4a	100	275	100	7.57/15.62	5.97/15.54	4	2	2	1
e^#^	gi/34925032	RecName: Full = Wheatwin-2; AltName:Full = Pathogenesis-related protein 4b	100	224	100	8.18/15.86	6.88/15.63	4	2	2	1

^**a**^Number of phosphorylation sites predicted by NetPhos 2.0 Server.

*Spot numbers correspond to those shown in Figure 2 and Figure 3.

^#^Newly identified phosphorylated protein spots.

Phosphorylated modification sites on serine, threonine and tyrosine were predicted by NetPhos 2.0 Serve [35,36]. Generally, the predicted results were in accordance with those from Pro-Q Diamond dye staining (Table 2). For example, class II chitinase (spots 112, 113 and 114, gi/62465514) was predicted to possess 9 serine, 3 threonine and 1 tyrosine phosphorylated modification sites, whereas peroxidase 1 (spots 122 and 123, gi/22001285) had 11, 5 and 2 phosphorylated modification sites of serine, threonine and tyrosine, respectively. Phosphorylated protein staining showed that both proteins had strong staining signals (Figure 5B).

Through sub-cellular localization of the identified proteins during grain development, a large number of proteins appeared to locate in the cytosol (Table 1). Most of the identified enzymes with higher abundance, and involved in glycolysis-the TCA pathway-nitrogen metabolism were located in the cytosol especially during the first two stages. Until stage III many storage proteins appeared on the endoplasmic reticulum (ER). Stress-related proteins were mainly located in the plastids and were extracellular at all development stages. Additionally, some enzymes involved in starch synthesis also appeared in plastids during the early development stages.

### Protein expression profiles during grain development

**Figure 6:**
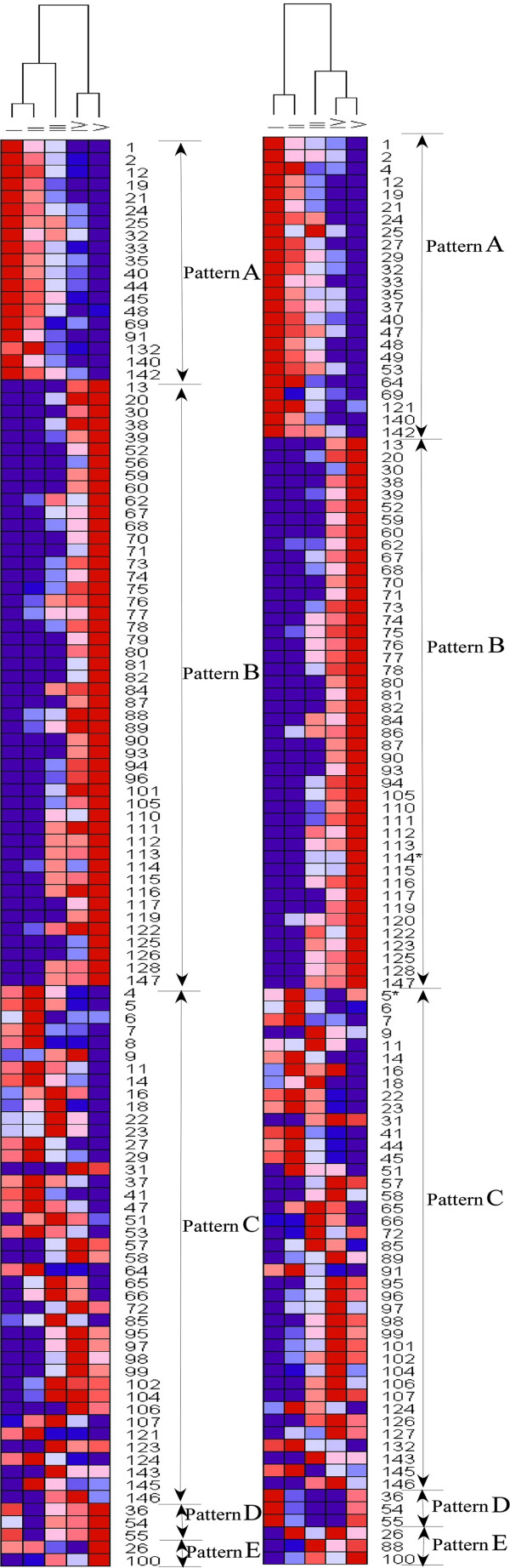
**Hirerarchical clustering analysis of differentially accumulated protein spots in Jimai 20 (left) and Zhoumai 16 (right).** Red color: the higher abundance of protein spots; blue color: the lower abundance of protein spots.

**Figure 7:**
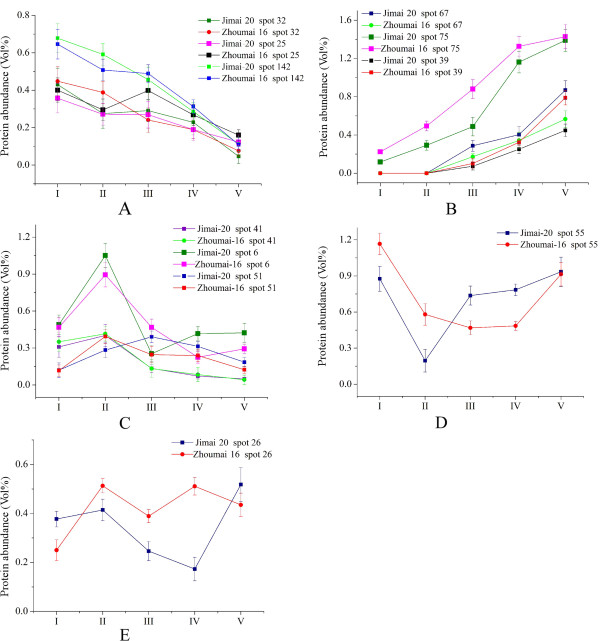
Protein expression patterns in Jimai 20 and Zhoumai 16 during five grain developmental stages (A-E).

The expression profiles of the 117 protein spots were investigated by hierarchical cluster analysis (Figure 6). Five main expression patterns (A-E) were present and clearly reflected three distinct grain development phases: differentiation (I-II), grain filling (II-IV) and desiccation/maturation (IV-V) as shown in Figure 6 and 7.

Expression pattern A included 19 spots in Jimai 20 and 24 in Zhoumai 16 exhibiting down-regulated modes during grain development. For example, triticin precursor (spot 69) was highly accumulated in the first two stages but was down-regulated at grain filling and maturity. Most of the proteins involved in the TCA pathway and glycolysis showed this expression pattern, such as isocitrate dehydrogenase NADP-dependent and its precursor (spots 32 and 33), and phosphoglycerate mutase (spots 1 and 2). Preprotein of glucose-1-phosphate adenylyltransferase (spot 35) also displayed this pattern in both cultivars.

Expression pattern B included the largest proportion of identified proteins and showed up-regulated expression, especially during the late grain development stages. Totals of 48 spots in Jimai 20 and 44 in Zhoumai 16 were in this expression group, which contained most of the storage proteins, many stress/defense-related proteins and two enzymes involved in alcoholic fermentation. In general, storage proteins including globulins (spots 70, 71, 80 and 82), gliadins (spots 74, 75, 76 and 77) and glutenins (spot 78), triticins (spots 67 and 68) and avenin-like protein (spot 73) accumulated significantly at the later developmental stages, but only had trace expression levels during the earlier developmental stages in both cultivars. The same responses also occurred for formate dehydrogenase (spot 38 and 39) involved in alcoholic fermentation, and class II chitinase (spots 112–116) involved in stress/defense pathways. Three spots (121, 122 and 123) were identified as peroxidase 1, of which 122 displayed B expression pattern in both cultivars.

Expression pattern C was the second largest group of identified proteins, represented by 41 spots in Jimai 20 and 40 in Zhoumai 16, many of which included glycolysis and stress/defence-related proteins and showed up-regulated expression at the early development stages, and were then down-regulated with advancement of grain filling and maturity. Representative proteins involved in this expression group were phosphoglucomutase (spot 18) and glucose-1-phosphate adenylyltransferase (spot 41) related to starch synthesis.

Expression pattern D, unlike pattern C, displayed a down to up-regulated expression trend. Only spots 36, 54 and 55 representing two proteins (peptidylprolyl isomerase Cyp2 and cyclophiliwere) showed this pattern. The remaining two spots (26 and 100) in Jimai 20 and three (26, 88 and 100) in Zhoumai 16 accumulated a complicated pattern E that fluctuated during gain development (Figure 6 and 7).

### Comparative proteome characterization in Jimai 20 and Zhoumai 16 during grain development

Comparative proteomic analysis demonstrated a considerable proteome expression difference between Jimai 20 and Zhoumai 16 during grain development. A total of 20 protein spots co-accumulated in both wheat cultivars, but with different expression patterns (Figure 6). For example, starch-rating enzyme glucose-1-phosphate adenyl-transferase (AGPase, spots 44 and 45) displayed A expression patterns in Jimai 20, but C patterns in Zhoumai 16. Spots 121 and 123 were identified as peroxidase I that showed expression pattern C in Jimai 20, and patterns A and B in Zhoumai 16. In addition, 27 K protein (spot 86) and superoxide dismutase (spot 127) gradually accumulated in Zhoumai 16, but showed little change in Jimai 20 during all five stages.

**Figure 8:**
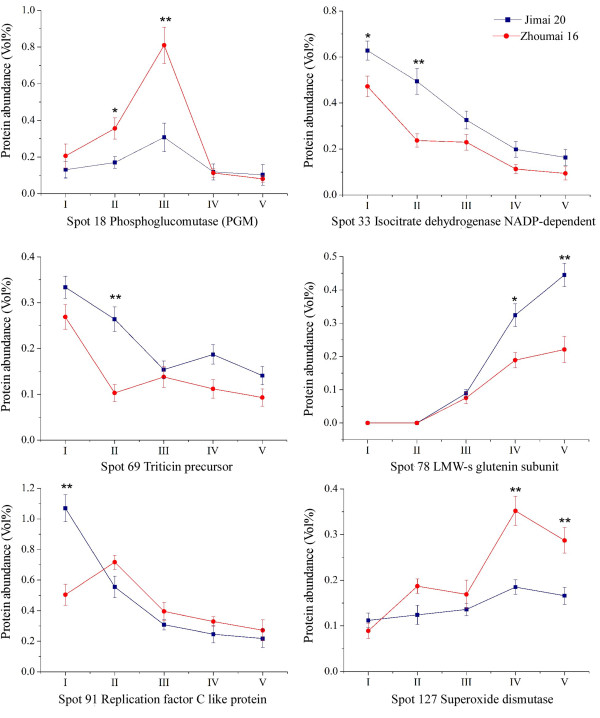
**Differential expression of six protein spots in Jimai 20 and Zhoumai 16 during five grain development stages.** Spot 18 (II/III); spot 33 (II); spot 69 (II); spot 78 (V); spot 91 (I); spot 127 (IV).

Protein spots with two fold changes, or greater, in abundance at particular times between the two cultivars were considered to be cultivar-different proteins. Twenty seven spots showed cultivar-different during the five development stages and they were mainly involved in carbohydrate metabolism, stress/defense, protein storage and transcription/translation. Among them, spots 8, 56 and 79 (glyceraldehyde-3-phosphate dehydrogenase, cyclophilin and S-type low molecular weight glutenin L4-55) only accumulated in Jimai 20, but were not detected in Zhoumai 16 when subjected to ImageMaster™ 2D Platinum Software analysis. Another two spots, 49 and 120 (aspartate aminotransferase and monomeric alpha-amylase inhibitor), only accumulated in Zhoumai 16. In the remaining protein spots, 14 accumulated in higher abundance in Jimai 20 than in Zhoumai 16, whereas 8 were more abundantly accumulated in Zhoumai 16. For example, important proteins displaying higher levels of expression in Jimai 20 than in Zhoumai 16 included isocitrate dehydrogenase NADP-dependent (spot 33), triticin precursor (spot 69), LMW-s glutenin subunit (spot 78), and replication factor C like protein (spot 91) as shown in Figure 8. Some proteins displayed higher expression abundances in Zhoumai 16 than in Jimai 20, for example, phosphoglucomutase (spots 18 and 19).

### Transcriptional expression analysis by qRT-PCR

**Figure 9:**
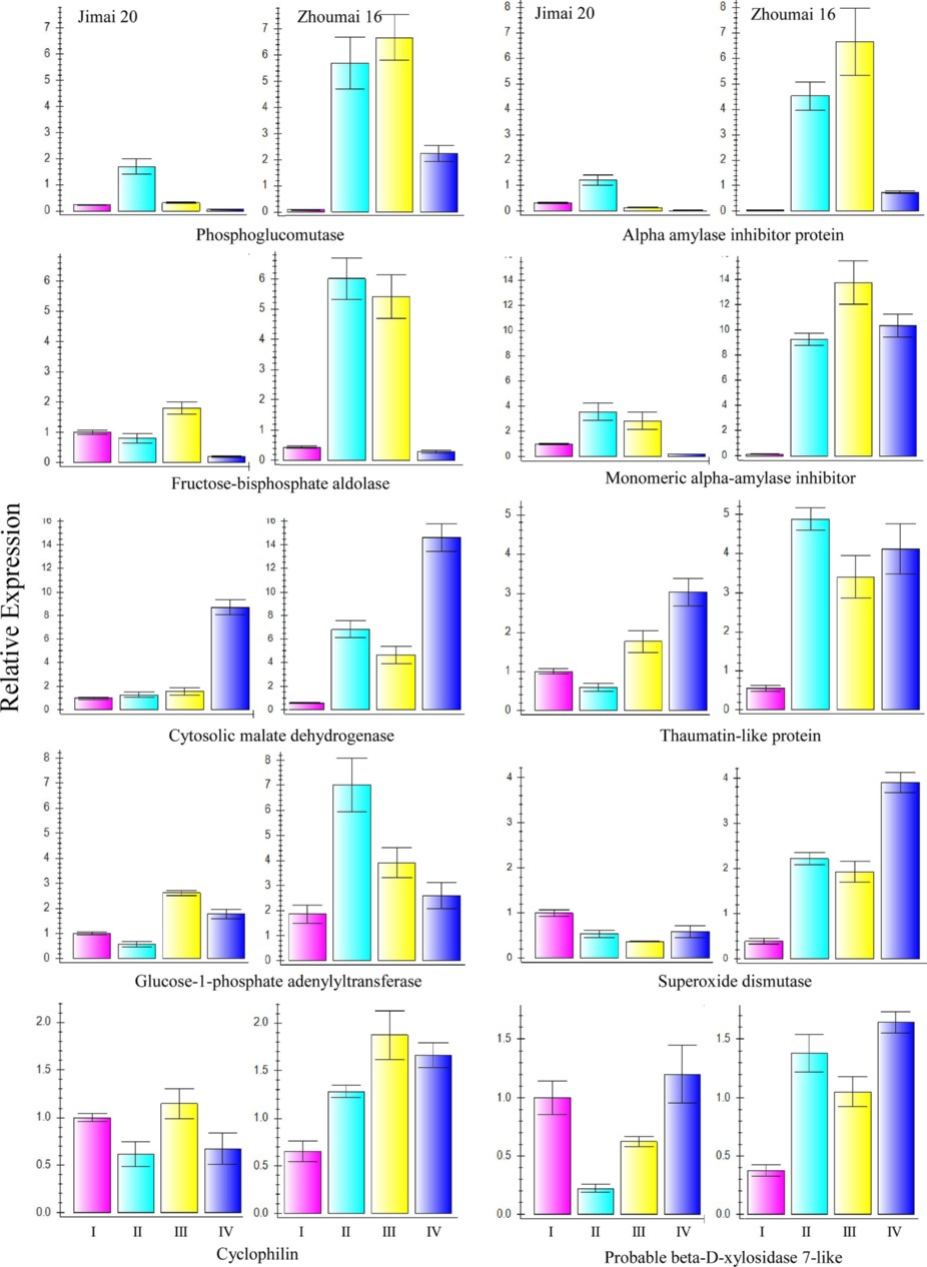
Transcriptional expression profiles of 10 representative protein genes from four grain development stages (I-IV) determined by qRT-PCR.

Since wheat grains in stage V were approaching maturity and the reference gene accumulated unstably, the transcriptional expression profiles of ten representative genes from stages I to IV were investigated by qRT-PCR with specific primers ( Additional file 3: Table S3). As shown in Figure 9, the transcriptional expression patterns of only four protein genes (phosphoglucomutase, thaumatin-like protein, superoxide dismutase and monomeric alpha-amylase inhibitor) in both cultivars and two genes (glucose-1-phosphate adenylyltransferase and alpha amylase inhibitor protein) in Zhoumai 16 were consistent with their protein expression models. The remaining protein genes showed poor consistency between their transcriptional and translational levels. Interestingly, alpha-amylase inhibitor was shown to be phosphorylated when stained by Pro-Q Diamond dye (Table 2), a feature that might be responsible for lower consistency between transcriptional and translational expression patterns.

## Discussion

In this study samples were collected during grain development and categorized according to cumulative average daily temperatures to avoid the effects of temperature on plant growth and development [37]. Proteome expression profiles during five grain development stages in two bread wheat cultivars were investigated by 2-DE and MALDI-TOF/TOF-MS. The results revealed key molecular characteristics of grain development and provided important insights on crucial proteins and their expression profiles during specific developmental phases.

### Metabolism and energy supply

The respiratory pathways of glycolysis, the tricarboxylic acid (TCA) cycle and the mitochondrial electron transport chain are essential for energy provision in a wide range of cellular functions [38]. We identified 6 spots representing 4 unique proteins related to the TCA cycle, including NAD-dependent isocitrate dehydrogenase (NAD-IDH) and NAD-IDH precursor (spots 33 and 32). NAD-IDH, present in mitochondria, is a key enzyme in the TCA cycle and catalyzes the oxidative decarboxylation of isocitrate towards 2-oxoglutarate, NADH, and CO_2_. The TCA cycle-related proteins all showed high expression levels from 6 to 15 DAP (147-353^o^Cd), and then underwent steep decreases. This implies that the TCA cycle is very active during the early grain development stages, mainly providing energy for grain development. NAD-IDH expression was higher in Jimai 20 than in Zhoumai 16 from 6 to 11 DAP, especially at 11 DPA. However, some other energy-related enzymes were accumulated as cultivar-different spots, such as phosphoglucomutase (PGM, spots 18 and 19), which were highly accumulated in Zhoumai 16. These differential expressions in energy metabolism in different genotypes were possibly related to different cultivar performances.

For glycolysis 21 spots representing 14 unique proteins were identified. Except for a few spots such as PGM (spots 18 and 19), the vast majority of glycolysis-related proteins showed simultaneous peaks in expression around 11 DPA (252^o^Cd) in both cultivars. This stage is consistent with cell differentiation and the beginning of dry matter accumulation during which lots of energy is needed [14]. At the same time, B-type starch granules began to form and enlarge (Figure 1C), resulting in decreased O_2_ availability and increased energy need. Therefore, glycolysis becomes a highly significant source of energy for grain filling, which involves packaging of proteins as well as starch [16]. PGM was accumulated at higher levels during early grain development, and showed a peak at 15 DAP (353^o^Cd), before decreasing to a lower constant level. This coincided with an intensive starch synthesis stage [14]. Previous reports demonstrated that PGM, as a phosphorylated protein, catalyses the bidirectional interconversion of glucose 1-phosphate (G1P) and glucose 6-phosphate (G6P) and cytosolic PGM isoforms may play an important role in the partitioning of carbon between the starch synthetic (G1P-utilizing) and glycolytic (G6P-utilizing) pathways in plant tissues [39,40]. Phosphorylation of starch-related enzymes in wheat and maize, such as GBSS, BEIIb, SSIIa and starch phosphorylase appeared to play important roles in synthesis and accumulation, and protein phosphorylation in wheat amyloplasts could regulate starch branching enzyme activity and protein–protein interactions [28,41,42]. Our results also showed that PGM was a phosphorylated protein by Pro-Q Diamond dye detection (Figure 5B), indicating its importance in glycolysis and energy provision for starch synthesis. Additionally, the expression abundance of PGM was higher in Zhoumai 16 than Jimai 20 during the early grain development stages (Figure 8), and this might be partly responsible for greater grain size and higher yield of Zhoumai 16 than Jimai 20.

Nucleoside diphosphate kinase (NDPK) is a ubiquitous enzyme functioning in intracellular distribution of terminal phosphate bond energy among the various nucleotides used in synthesis pathways and regulatory functions in cells [43,44]. Plant NDPKs are also involved in signal transduction, differentiation and development [45-47]. Our results showed that NDPK was gradually down-regulated during grain development in both wheat cultivars (Figure 9), and therefore might play an important role in signal regulation for grain development.

Glycolysis and the TCA cycle not only provide energy and intermediates for synthesis of metabolites, but their metabolite concentration gradients may also act as signals for the onset of the seed maturation phase and its regulation [11,48]. During the late development stages of wheat grains, the alcoholic fermentation pathway could contribute supplementary energy in anaerobic conditions due to an energy deficit from glycolysis and the TCA cycle. In addition, formate dehydrogenase showed increased expression at stage III (15 DAP) and further increased during the late grain filling and desiccation stages. This is consistent with the transition from cell growth and differentiation to starch synthesis. A switch from central carbon metabolism to alcoholic fermentation may be important for starch synthesis and accumulation during grain development [12].

### Starch synthesis

Grain yield is largely determined by starch accumulation during the grain filling phase in cereals [49]. Starch biosynthesis is initiated with a substrate of ADP-glucose formed by glucose-1-phosphate adenylyltransferase (ADP-glucose pyrophosphorylase/AGPase) from glucose-l-phosphate. AGPase, located in plastids, is a major rate-limiting enzyme in seed starch biosynthesis [50]. Our results demonstrated that glucose-1-phosphate adenylyltransferase large subunits (spots 41, 44 and 45) showed the highest expression levels from 6 to 11 DPA (147-252^o^Cd), and was then down-regulated to trace levels at grain desiccation. The expression pattern closely matched large increases in both starch content and grain weight. Plastid AGPase activity maintains higher levels from 8 to 17 DPA and reaches a peak around 11 DPA, before declining at the later stages of endosperm development [51]. By coincidence ADP-glucose pyrophosophorylase preprotein (spot 35), the precursor of AGPase in plastids, showed peak expression at 147^o^Cd (6 DPA), and was then down-regulated simultaneously in both cultivars. The increase in AGPase could result in reduced ADP-glucose pyrophosophorylase preprotein during grain development.

### Stress and defense

The accumulation of starch and storage proteins was accompanied by the expression of various α-amylase inhibitors, which are mainly located in plastids or in extracellular spaces. Alpha-amylase inhibitors play important roles in protecting starch and protein reserves in the endosperm against degradation, particularly that caused by biotic stresses such as insect attack [16,52]. We identified 11 α-amylase inhibitors (spots 94–102, 119 and 120) and all accumulated at low levels during early grain development, but were rapidly up-regulated from the starch synthesis to grain desiccation stages in both wheat cultivars, consistent with the accumulation patterns of starch and storage proteins during late grain development. Twenty-one spots were identified as α-amylase inhibitors, predominant proteins in wheat flour [19]. Probably due to protein PTMs, the up-regulation of some α-amylase inhibitors and the down-regulation of other α-amylase inhibitors may reflect their different roles in high temperature treatments or drought stress [53].

Chitinases, mainly located extracellularly, catalyse the hydrolytic cleavage of the β-1, 4-glycoside bonds present in biopolymers of N-acetylglucosamine in chitin, a major component of cell walls in fungi. Some reports showed that transgenic wheat expressing a barley chitinase exhibit enhanced resistance against powdery mildew, leaf rust and *Fusarium* head blight [54-56]. In our study, five protein spots were identified as class II chitinases; all exhibited trace expression levels during early grain development, but accumulated during grain filling in both cultivars. Chitinases (spots 112, 113 and 114) with different molecular weights or isoelectric points were also shown to be phosphorylated, consistent with predictions from NetPhos 2.0 Serve and Pro-Q, using Diamond dye staining (Table 2 and Figure 5B). Many isoforms of chitinase also appeared in other plant species such as *Vicia faba*, *Pisum sativum*, *Hordeum vulgare*, *Zea mays* and *Glycine max*[57]*.* Protein modifications resulting from phosphorylation have been associated with biotic and abiotic stresses such as light, pathogen invasion, hormones, temperature stresses, and nutrient deficiencies [58-60]. Transient phosphorylated modifications of chitinases might play important roles in different stress/defense and/or detoxification mechanisms through regulation of their localization and enzyme activities.

Peroxidases are one kind of antioxidant enzymes involved in defense. Some investigations showed that peroxidase activity in whole wheat grains varied with development stage [61,62]. Plant tissues typically contain several peroxidase isoforms varying in isoelectric point values from acidic to basic [63]. In our work, three spots (spots 121, 122 and 123) were simultaneously identified as peroxidase 1 with the same molecular mass but different isoelectric points. Spots 122 and 123 were both phosphorylated (Figure 5B). Interestingly, these peroxidases exhibited different expression patterns, where spot 121 was highly abundant during phases I and II but spots 122 and 123 showed only trace expression at these stages. Spots 122 and 123 were sharply up-regulated during the following grain filling phases. Differences in peroxidase activity were also observed in developing pea seedlings [64]. This suggests that peroxidase 1 may be involved in different functions through different expression patterns or phosphorylated modification. Another antioxidant enzyme is superoxide dismutase, catalyzing the first step in the active oxygen species scavenging system [65]. This enzyme was up-regulated gradually in Zhoumai 16 whereas it maintained a stable expression level in Jimai 20 during grain development, demonstrating a significant differential expression pattern in different wheat genotypes.

### Storage proteins and nitrogen metabolism

Storage proteins are major components of wheat gluten and are closely related to processing quality. In the current study five kinds of storage proteins identified during grain development included triticins, globulins, avenin-like proteins, γ-gliadins and LMW-s glutenin subunits. Four γ-gliadins (spots 74, 75, 76 and 77) were initiated from 6 DPA (147^o^Cd), and then sharply up-regulated at 20 DPA (461^o^Cd) and remained at maximum levels until grain maturity. However, globulins (spots 70, 71, 80 and 82), avenin-like protein (spot 73) and two kinds of LMW-s glutenin subunits (spots 78 and 79) were accumulated at the later developmental stages in both wheat cultivars. The abundance of the two LMW-s glutenin subunits was higher in Jimai 20 than in Zhoumai 16 at maturity, a feature possibly related to the superior gluten quality of Jimai 20.

Triticin, a minor storage protein in the starchy endosperm of wheat, is considered to be nutritionally rich due to the presence of a unique lysine-rich decapeptide repeat motif [66,67]. It is also thought to be involved in determination of flour quality because of the thiol disulfide interchange which takes place during grain maturation when subjected to dehydration stress [66]. Our results showed that the expression of triticins began at 15 DPA (353^o^Cd) and reached a maximum that lasted until maturity in both cultivars. Previous work showed that triticins were more highly accumulated in superior quality wheat varieties than in lower quality genotypes [15,68,69]. In our study, triticin (spot 67) showed a slightly higher expression in Jimai 20 than in Zhoumai 16 (Figure 7). Conversely to triticin expression, triticin precursor underwent a down-regulated expression pattern in both cultivars, indicating that triticin precursor was probably consumed and converted to the accumulating triticin during grain filling. Triticin precursor also accumulated at a higher level in Jimai 20 than in Zhoumai 16 during early grain development, especially at 11 DPA (252^o^Cd) (Figure 8). This might relate to the superior quality of Jimai 20. One triticin spot was phosphorylated (Table 2). A recent report also identified seven protein spots from white flour of cv Butte 86 as triticin that had undergone post-translational modifications [19].

Generally, biosynthesis of seed storage protein is dependent on amino acid synthesis and transport of nitrogen metabolism [70]. Amino acids for protein synthesis are imported from leaves and stems by the phloem to the endosperm cavity [49]. In our study, many aminotransferases of amino-acids such as aspartate, alanine and serine as well as one putative methionine synthase were identified. These enzymes were abundant pre-storage phase from flowering to 15 DPA (353^o^Cd) and then declined with grain maturity in both wheat cultivars.

### Other functional proteins

Replication factor C (RFC), composed of one large subunit and four small subunits, is an important factor involved in DNA replication and repair mechanisms as well as cell proliferation [71]. RFC mediates genomic stability and transcriptional gene silencing in Arabidopsis [72]. They express strongly in proliferating tissues, such as shoot apical meristems (SAM) and developing grain [73]. In the present study, RFC-like protein (spot 91) accumulated in highest abundance at 6 DPA (147^o^Cd), and then displayed down-regulated expression during subsequent grain development stages in Jimai 20, whereas it showed peak expression at 11 DPA (252^o^Cd) in Zhoumai 16, demonstrating its expression differences in different genotypes. Tri-snRNP-specific 27 K protein is potentially involved in non-snRNP splicing factors-medicated protein/protein interactions and can be phosphorylated in vitro to modulate pre-mRNA splicing at the transcriptional level [74]. We identified 4 spots as 27 K proteins, of which 3 (87, 88 and 90) were up-regulated during grain development in both cultivars. The remaining spot (86) accumulated gradually in Zhoumai 16, whereas there was little change in Jimai 20 throughout grain development. WRKY is a relatively complex transcription factor family involved in transcriptional regulation and many other plant processes including responses to biotic and abiotic stresses [75]. In our work WRKY transcription factor was continuously up-regulated in both wheat cultivars until grain maturity, indicating importance during the grain development process.

## Conclusions

We used proteomic approaches to characterize protein accumulation during grain development in Chinese bread wheat cultivars Jimai 20 and Zhoumai 16, which differ in gluten quality. Among 117 differentially accumulated protein spots and 82 unique proteins, there were five main expression patterns, and a considerable number of proteome expression differences occurred between the two cultivars. Some cultivar-different protein spots in Jimai 20 accumulated in higher abundance than in Zhoumai 16, such as triticin precursor and LMW-s glutenin subunit, might be related to superior gluten quality. In addition, some proteins with different isoforms in developing grains appeared to be phosphorylated, a feature that which could play important roles in wheat grain development, and possibly lead to expression differences between proteins and their mRNA. Our results provide new insights into proteome characterization during grain development in different wheat genotypes.

## Methods

### Plant materials

Bread wheat (*Triticum aestivum* L., 2n = 6x = 42, AABBDD) cultivars Jimai 20 and Zhoumai 16 were planted at the experimental station of the Chinese Academy of Agricultural Science (CAAS), Beijing, during the 2010–2011 growing season (October-June). Both cultivars have almost the same growing, flowering and mature times, and their main differences are gluten quality properties, viz. Jimai 20 with superior and Zhoumai 16 with poor gluten quality [32].

Wheat plants were grown under the same natural soil conditions, fertilized (130 kg N-ha^-1^) and watered as usual. According to the method of Majoul et al. [37] grain samples were harvested during the post-anthesis period based on thermal times corresponding to the following cumulative average daily temperatures (°Cd): 147°Cd (I), 252°Cd (II), 353°Cd (III), 461°Cd (IV) and 749°Cd (V) as shown as Table 3. Sampled grains from three replicates were collected and stored at −80°C prior to analysis.

**Table 3:** Details of grain samples harvested during the post-anthesis period based on thermal time corresponding to cumulative average daily temperatures

**Batch/No.**	**Date**	DPA*	**°Cd**
I	2011.05.04-05.10	6	147°C
II	2011.05.10-05.15	11	252°C
III	2011.05.15-05.19	15	353°C
IV	2011.05.19-05.24	20	461°C
V	2011.05.24-06.05	31	749°C

*Days post-anthesis.

### Scanning electronic microscope (SEM) studies of endosperm

The dissection of developing grains was undertaken using a scanning electronic microscope (SEM) S-4800 FESEM (Hitachi, Japan) and the average fresh seed weight was measured. Grain samples from five stages of development were fixed in aqueous solutions containing 44.5% ethanol, 1.85% methanol and 6% glacial acetic acid for more than 24 h, and then transferred into 70% ethanol and stored at 4°C prior to SEM observations. Samples were dehydrated stepwise in ethanol of ascending concentrations including 70% (15 min), 80% (15 min), 90% (overnight) and 100%. The endosperms were then treated stepwise for 15 min in mixtures of ethanol and isoamyl acetate with ratios 3:1, 1:1 and 1:3, and were finally soaked in isoamyl acetate for 80 min. Finally, after critical point drying, they were observed.

### Protein preparation

Grain samples of 500 mg from the middle part of wheat ears were ground into meal in liquid nitrogen and then further ground for 7–8 min with 1 ml of extraction buffer (400 mM sucrose, 35 mM Tris–HCl, pH 7.5, 200 mM EDTA, l mM DTT and l mM PMSF), followed by 2–3 min in extraction buffer (400 mM sucrose, 35 mM Tris–HCl, pH 7.5, 200 mM EDTA, 400 mM Triton X-100, l mM DTT and l mM PMSF). Samples were shaken vigorously for 15 min at room temperature. After twice centrifuging for 15 min at 13000 rpm, the supernatants were transferred to new tubes. Protein supernatants were precipitated with 1/4 volumes of cold 10% trichloroacetic acid at −20°C for 2–3 h. After centrifuging for 5 min at 13000 rpm, the pellets were rinsed with cold (−20°C) acetone, and then thrice centrifuged at 8000 rpm for 10 min. After freeze-drying, the pellets were added to 300 μl of solubilisation buffer (7 M urea, 2 M thiourea, 4% CHAPS) at the room temperature for 2 h. After removal of insoluble material by centrifuging for 10 min at 13000 rpm, the concentrations of protein samples were determined with a 2-D Quant Kit (Amersham Bioscience, USA).

### 2-DE and data analysis

Protein samples of 600 μg were loaded onto Immobiline DryStrip (GE Healthcare, 18 cm, pH 3–10 L, USA) by an Ettan^TM^ IPG-phor II^TM^ system (GE Healthcare, USA). Rehydration and isoelectric focusing, equilibration of strips and SDS-PAGE were performed as described earlier [76]. 2-DE experiments were repeated three times by using protein samples independently prepared from separate seed samples. Proteins were visualized by colloidal Coomassie Brilliant blue (CBB) staining (R-250/G-250 = 4:1) and analysis was performed by ImageMaster™ 2D Platinum Software Version 5.0 (GE Healthcare) following the procedures specified in the software. Firstly, spot were detected, quantified, and background were subtracted. For spot profile analysis, the first replication 2-DE gel of 6 DPA seed samples was selected as the reference gel. All analyzed gels were matched individually to the reference gel. Then, the spots that all existed in three independent sample sets were selected and all matched spots were checked manually. At last, differentially accumulated protein spots were determined according to statistically significant changes between samples by Student's *t*-test (abundance variation at least 2-fold, *p* < 0.05). Hierarchical cluster analysis was computed to analyze differentially accumulated protein spots for protein clustering according to Pearson’s distance ( http://www.broadinstitute.org/cancer/software/genepattern/).

### Detection of phosphorylated proteins

Protein samples were newly extracted adding PhosSTOP Inhibitor Cocktail Tablets (Roche) after 2-DE analysis described above. Gels were stained with Pro-Q Diamond (Invitrogen) according to the manufacturer’s instructions. Briefly, the gels were fixed twice in 250 ml of immobilisation solution (50% methanol and 10% acetic acid) for 30 min, and washed three times with 250 ml ddH_2_O for 10 min. The gels were incubated in Pro-Q Diamond stain in darkness for 2 h. To remove non-specific background material, the gels were thrice destained with 20% acetonitrile in 50 mM sodium acetate (pH 4.0) for 30 min, and then twice washed with 250 ml ddH_2_O for 5 min. Finally, the gels were scanned on a Typhoon^TM^ 9400 scanner (GE Healthcare, USA) with a 532 nm excitation laser and a 580 nm long pass filter with a resolution of 100 microns. After fluorescent image acquisition, the gels were stained with CBB to visualize total proteins.

### Protein identification through mass spectrometry

Differentially accumulated and phosphorylated protein spots were identified by matrix-assisted laser desorption/ionization time-of-flight tandem mass spectrometry (MALDI-TOF/TOF-MS) according to Guo et al. [76]. Briefly, selected protein spots were excised manually from the gels and destained with 100 mM NH_4_HCO_3_ in 30% acetonitrile (ACN) and lyophilized before digestion at 37°C overnight (16 h) with 20 μl of 50 mM NH_4_HCO_3_ containing 0.01 mg/ml sequencing grade modified trypsin (Promega, Madison, WI, USA). The peptides were three times extracted with 0.1% TFA in 60% ACN and tryptic peptides were then dissolved in 5 mg/ml cyano-4-hydroxycinnamic acid (CHCA) in 50% ACN and 0.1% trifluoroacetic acid (TFA). Identification of the tryptic peptides was performed using an ABI 4800 Proteomic Analyzer MALDI-TOF/TOF-MS (Applied Biosystems/MDS Sciex, USA). The MS together with MS/MS spectra were searched against the NCBI non-redundant viridiplantae database using softwares GPS Explorer (Applied Biosystems) and MASCOT (Matrix Science) with the following parameter settings: trypsin cleavage, two missed cleavages allowed, peptide mass tolerance set to ± 0.2 Da, fragment tolerance set to ± 0.3 Da. The proteins with Protein Score C.I. % and Total Ion Score C.I. % above 95% were identified as credible results for MS/MS.

### Prediction of subcellular localization

Subcellular locations of identified proteins were predicted using WoLF PSORT ( http://wolfpsort.org/) [77], Predotar ( http://urgi.versailles.inra.fr/predotar/predotar.html) [78] and UniprotKB ( http://www.uniprot.org/) database programs. Protein localizations at the subcellular level were established by similar results from at least two programs.

### qRT-PCR analysis

Wheat grains were ground into fine powder in liquid nitrogen and then incubated in 1 ml TRIzol reagent (Invitrogen). Total RNA was extracted according to ther manufacturer's instructions, but adding an additional chloroform extraction. Genomic DNA was removed by digesting each sample (10 μg of total RNA) with Rnase-free DNaseI (Promega) according to the manufacturer’s instructions. After digestion, a 10 μl RNA sample was dissolved for 30 min at the room temperature. First-strand cDNA was synthesized in a 20 μl volume containing 0.5 μl AMV reverse transcriptase (Promega), 0.5 μl RNase inhibitor (Promega), 1 μl oligo dT primer, 2 μl dNTP mixture, 4 μl MgCl_2_ (25 mM), 2 μl 10 × reverse transcriptase buffer and 4 μl RNA sample. The reaction mixture was incubated at 42°C for 60 min.

Gene-specific primers were designed using Primer 5.0 and their specificities were checked by the melting curves of the RT-PCR products. Quantitative real-time PCR (qRT-PCR) was performed in 20 μl volumes containing 10 μl 2 × SYBR® Premix Ex Taq™ (TaKaRa), 2 μl 50-fold diluted cDNA, 0.4 μl of each gene-specific primer and 7.2 μl ddH_2_O. PCR conditions were as follows: 95°C for 3 min, 45 cycles of 15 s at 95°C, 57°C for 15 s and 72°C for 20 s. Three replicates were used for each sample. Reactions were conducted in a CFX96 Real-Time PCR Detection System (Bio-Rad). All data were analyzed with CFX Manager Software (Bio-Rad).

## Abbreviations

2-DE: two-dimensional electrophoresis; G1P: glucose 1-phosphate; G6P: glucose 6-phosphate; MALDI-TOF/TOF-MS: matrix-assisted laser desorption/ionization time-of-flight tandem mass spectrometry; NDPK: Nucleoside diphosphate kinase; PGM: phosphoglucomutase; PTMs: post-translational modifications; qRT-PCR: real-time quantitative reverse transcriptional PCR; SEM: scanning electronic microscope.

## Competing interests

This manuscript has no any financial and non-financial competing interests.

## Authors’ contributions

GG, LD and YX carried out all experiments and data analysis. SS, GP, LX and HY performed the preparation of cDNA, qRT-PCR and bioinformatics analyses. YY conceived the study, planned experiments, and helped draft the manuscript. All authors read and approved the final manuscript.

## Supplementary Material

Additional file 1 The peptide sequences of proteins during wheat grain development identified by MS/MS. Click here for file

Additional file 2 The peptide sequences of phosphorylated proteins during wheat grain development identified by MS/MS. Click here for file

Additional file 3 Primer sequences used for quantitative real-time RT-PCR (qRT-PCR). Click here for file
