# Supplementary material for: Proteome characterization of developing grains in bread wheat cultivars (Triticum aestivum L.)
Source: BMC Plant Biol. 2012 Aug 19;12:147. doi: 10.1186/1471-2229-12-147 (PMC3480910; doi:10.1186/1471-2229-12-147)
Supplement: Additional file 1 — The peptide sequences of proteins during wheat grain development identified by MS/MS. [file 1471-2229-12-147-S1.doc]

**Appendix Table 1 The peptide sequences of proteins during wheat grain development identified by MS/MS**

| **Spot**  **No.** | **Accession**  **No.** | **Protein Name** | **±da** | **±ppm** | **Start Sequence** | **End Sequence** | **Peptide Sequence** | **Ion Score** | **Ion Score**  **C.I.%** |
| --- | --- | --- | --- | --- | --- | --- | --- | --- | --- |
| **Carbohydrate metabolism** | | | | | | | | | |
| **1. TCA pathway:** | | | | | | | | | |
| 22 | gi/49343245 | Cytosolic malate dehydrogenase | -0.0184 | -21 | 165 | 172 | ALGQISER | 21 | 67.85 |
|  |  |  | -0.0582 | -43 | 57 | 68 | MELIDAAFPLLK | 62 | 100 |
|  |  |  | -0.063 | -46 | 57 | 68 | MELIDAAFPLLK | 39 | 99.53 |
|  |  |  | -0.0461 | -28 | 242 | 257 | LSSALSAASSACDHIR | 39 | 99.48 |
|  |  |  | -0.0806 | -31 | 69 | 94 | GVVATTDVVEACTGVNV | 18 | 43.74 |
|  |  |  | -0.087 | -33 | 69 | 94 | GVVATTDVVEACTGVNV | 29 | 95.52 |
| 23 | gi/49343245 | Cytosolic malate dehydrogenase | -0.1044 | -77 | 57 | 68 | MELIDAAFPLLK | 29 | 94.48 |
|  |  |  | -0.098 | -71 | 57 | 68 | MELIDAAFPLLK | 45 | 99.84 |
| 24 | gi/37928995 | Cytosolic malate dehydrogenase | -0.0626 | -46 | 27 | 38 | MELIDAAFPLLK | 47 | 94.69 |
|  |  |  | -0.0818 | -34 | 183 | 202 | ELVQDDEWLNGEFIATVQQR | 157 | 100 |
|  |  |  | -0.0649 | -27 | 152 | 173 | NAIIWGNHSSSQYPDVNHATVK | 62 | 99.86 |
| 25 | gi/15232820 | MDH (malate dehydrogenase); malate dehydrogenase | -0.1122 | -92 | 224 | 234 | LFGVTTLDVVR | 51 | 99.09 |
|  |  |  | -0.1233 | -91 | 223 | 234 | KLFGVTTLDVVR | 50 | 98.90 |
| 32 | gi/7488742 | Isocitrate dehydrogenase (NADP) precursor | 0.0082 | 8 | 156 | 163 | HAFGDQYR | 53 | 99.93 |
|  |  |  | 0.0242 | 18 | 325 | 337 | TIEAEAAHGTVTR | 53 | 99.53 |
|  |  |  | 0.0171 | 12 | 262 | 272 | YEAAGIWYEHR | 37 | 79.64 |
| 33 | gi/92875135 | Isocitrate dehydrogenase (NADP+) | -0.0194 | -20 | 135 | 142 | HAFGDQYR | 36 | 32.01 |
|  |  |  | -0.0218 | -14 | 239 | 251 | SKYEAAGIWYEHR | 48 | 95.99 |
| **2.Glycolysis:** | | | | | | | | | |
| 1 | gi/32400802 | Phosphoglycerate mutase | 0.0983 | 66 | 140 | 151 | ALEFPDFDKFDR | 27 | 91.95 |
|  |  |  | 0.0954 | 53 | 114 | 130 | SVGPIVDGDAVVTFNFR | 31 | 96.68 |
| 2 | gi/32400802 | Phosphoglycerate mutase | 0.0596 | 50 | 175 | 184 | YLVSPPLIER | 34 | 69.10 |
|  |  |  | 0.0566 | 38 | 140 | 151 | ALEFPDFDKFDR | 71 | 100 |
| 4 | gi/120680 | Glyceraldehyde-3-phosphate dehydrogenase, cytosolic | -0.0748 | -50 | 237 | 250 | VPTVDVSVVDLTVR | 102 | 100 |
|  |  |  | -0.0588 | -35 | 68 | 82 | TLLFGEKPVTVFGVR | 72 | 99.99 |
|  |  |  | -0.0376 | -17 | 274 | 293 | GIMGYVEEDLVSTDFVGDSR | 128 | 100 |
|  |  |  | -0.0523 | -17 | 274 | 293 | GIMGYVEEDLVSTDFVGDSR | 96 | 100 |
| 5 | gi/120680 | Glyceraldehyde-3-phosphate dehydrogenase, cytosolic | -0.0665 | -44 | 237 | 250 | VPTVDVSVVDLTVR | 88 | 100 |
|  |  |  | -0.0472 | -28 | 68 | 82 | TLLFGEKPVTVFGVR | 68 | 99.84 |
|  |  |  | -0.0348 | -19 | 312 | 325 | LVSWYDNEWGYSNR | 98 | 100 |
|  |  |  | 0.0005 | 0 | 274 | 293 | GIMGYVEEDLVSTDFVGDSR | 127 | 100 |
| 6 | gi/120668 | Glyceraldehyde-3-phosphate dehydrogenase, cytosolic | -0.0376 | -40 | 43 | 50 | EVAVFGCR | 51 | 99.12 |
|  |  |  | -0.0801 | -71 | 17 | 25 | YDTVHGQWK | 44 | 95.43 |
|  |  |  | -0.062 | -41 | 205 | 218 | VPTVDVSVVDLTVR | 78 | 100 |
|  |  |  | -0.0375 | -21 | 280 | 293 | LVSWYDNEWGYSTR | 96 | 100 |
| 7 | gi/148508784 | Glyceraldehyde-3-phosphate dehydrogenase | -0.0398 | -42 | 75 | 82 | EVAVFGCR | 18 | 20.37 |
|  |  |  | -0.0631 | -46 | 129 | 140 | DAPMFVCGVNEK | 20 | 43.88 |
|  |  |  | -0.07 | -47 | 237 | 250 | VPTVDVSVVDLTVR | 33 | 97.21 |
|  |  |  | -0.1093 | -49 | 274 | 293 | GILGYVDEDLVSTDFQGD | 28 | 91.68 |
| 8 | gi/148508784 | Glyceraldehyde-3-phosphate dehydrogenase | 0.0202 | 22 | 75 | 82 | EVAVFGCR | 17 | 23.26 |
|  |  |  | 0.0074 | 5 | 129 | 140 | DAPMFVCGVNEK | 48 | 99.95 |
|  |  |  | 0.0207 | 14 | 237 | 250 | VPTVDVSVVDLTVR | 42 | 99.76 |
|  |  |  | 0.0213 | 10 | 274 | 293 | GILGYVDEDLVSTDFQGD | 19 | 52.58 |
| 9 | gi/148508784 | Glyceraldehyde-3-phosphate dehydrogenase | -0.0016 | -1 | 237 | 250 | VPTVDVSVVDLTVR | 51 | 99.97 |
|  |  |  | -0.0139 | -6 | 274 | 293 | GILGYVDEDLVSTDFQGD | 75 | 100 |
| 11 | gi/18978 | Glyceraldehyde 3-phosphate dehydrogenase | 0.0188 | 16 | 49 | 57 | YDTVHGHWK | 43 | 85.65 |
|  |  |  | 0.0457 | 27 | 68 | 82 | TLLFGEKPVTVFGVR | 64 | 99.89 |
|  |  |  | 0.0691 | 39 | 312 | 325 | LVSWYDNEWGYSNR | 100 | 100 |
|  |  |  | 0.0901 | 41 | 274 | 293 | GDSIMRGYVEEDLVSTDFVG | 145 | 100 |
| 12 | gi/18978 | Glyceraldehyde 3-phosphate dehydrogenase | -0.0666 | -44 | 237 | 250 | VPTVDVSVVDLTVR | 131 | 100 |
| 13 | gi/7579064 | Cytosolic glyceraldehyde-3-phosphate Dehydrogenase GAPDH | -0.0052 | -3 | 135 | 148 | VPTVDVSVVDLTVR | 41 | 81.59 |
|  |  |  | 0.0049 | 3 | 210 | 223 | LVSWYDNEWGYSTR | 85 | 99.99 |
|  |  |  | 0.0293 | 13 | 172 | 191 | GILGYVDEDLVSTDFQGDNR | 112 | 100 |
| 14 | gi/32478662 | Cytosolic glyceraldehyde-3-phosphate dehydrogenase | -0.0503 | -34 | 68 | 81 | VPTVDVSVVDLTVR | 76 | 100 |
|  |  |  | -0.0063 | -3 | 105 | 124 | GILGYVDEDLVSTDFQGDNR | 116 | 100 |
| 16 | gi/28172909 | Cytosolic 3-phosphoglycerate kinase | -0.0271 | -16 | 32 | 48 | LAAALPDGGVLLLENVR | 32 | 97.20 |
|  |  |  | -0.0265 | -14 | 64 | 81 | LASVADLYVNDAFGTAH | 21 | 64.15 |
| 18 | gi/18076790 | Phosphoglucomutase (PGM) | -0.0412 | -24 | 259 | 274 | EDFGGGHPDPNLTYAK | 32 | 97.51 |
|  |  |  | -0.0443 | -23 | 285 | 303 | TSNVEPPEFGAAADGDA | 26 | 91.83 |
| 19 | gi/18076790 | Phosphoglucomutase (PGM) | -0.1393 | -127 | 206 | 214 | TIFDFESIK | 47 | 99.97 |
| 20 | gi/226316441 | Fructose-bisphosphate aldolase | -0.0264 | -18 | 40 | 52 | FASINVENVEDNR | 27 | 91.57 |
|  |  |  | -0.0324 | -19 | 40 | 53 | FASINVENVEDNRR | 22 | 75.75 |
| 21 | gi/18496065 | Putative fructose 1-,6-biphosphate aldolase | -0.0997 | -48 | 58 | 77 | IAGRATEPSQLSIDQNAQGL | 84 | 99.19 |
| 26 | gi/1785948 | Cytosolic triosephosphate isomerase | -0.0301 | -32 | 5 | 12 | FFVGGNWK | 55 | 99.19 |
|  |  |  | -0.0354 | -22 | 176 | 190 | VATPAQAQEVHANLR | 93 | 100 |
| 27 | gi/11124572 | triosephosphat-isomerase | 0.0355 | 37 | 5 | 12 | FFVGGNWK | 57 | 99.57 |
|  |  |  | 0.0675 | 48 | 101 | 113 | SLMGESSEFVGEK | 59 | 99.75 |
| 29 | gi/11124572 | Triosephosphat-isomerase | -0.0542 | -57 | 5 | 12 | FFVGGNWK | 55 | 99.03 |
|  |  |  | -0.0748 | -53 | 101 | 113 | SLMGESSEFVGEK | 66 | 99.93 |
|  |  |  | -0.0724 | -46 | 176 | 190 | VASPAQAQEVHANLR | 114 | 100 |
| 37 | gi/133872550 | Bp2A protein | -0.0942 | -97 | 172 | 179 | SGYFDETR | 20 | 79.92 |
|  |  |  | -0.1535 | -102 | 94 | 105 | ALEFPDFDKFDR | 48 | 99.96 |
|  |  |  | -0.1985 | -111 | 68 | 84 | SVGPIVDGDAVVTFNFR | 52 | 99.99 |
| **3. Alcoholic fermentation:** | | | | | | | | | |
| 38 | gi/32400847 | Formate dehydrogenase | 0.0829 | 112 | 171 | 176 | ILILLR | 17 | 44.75 |
|  |  |  | 0.1655 | 106 | 45 | 58 | IVGVFYQAGEYADK | 51 | 99.98 |
| 39 | gi/32400847 | Formate dehydrogenase | -0.0459 | -62 | 171 | 176 | ILILLR | -0.0459 | 42.37 |
|  |  |  | -0.1295 | -83 | 45 | 58 | IVGVFYQAGEYADK | -0.1295 | 99.81 |
|  |  |  | -0.1145 | -71 | 59 | 73 | NPNFVGCVEGALGIR | -0.1145 | 99.99 |
| **4. Starch and polysaccharide metabolism:** | | | | | | | | | |
| 35 | gi/21680 | ADP-glucose pyrophosophorylase preprotein | 0.0637 | 39 | 302 | 315 | YAELHDFGSEILPR | 39 | 81.28 |
|  |  |  | 0.0713 | 41 | 189 | 203 | SIEHILILSGDQLYR | 34 | 45.63 |
| 41 | [Q0PG36](http://www.agbase.msstate.edu/cgi-bin/getEntry.pl?db_pick=all&database=UniProtKB/TrEMBL&uid=Q0PG36_WHEAT&gb_acc=Q0PG36) | Glucose-1-phosphate adenylyltransferase | -0.0093 | -10 | 380 | 387 | TPFFTSPR | 18 | 31.99 |
|  |  |  | -0.0169 | -13 | 111 | 122 | ATPAVPIGGCYR | 21 | 70.59 |
|  |  |  | -0.0252 | -15 | 321 | 334 | YAELHDFGSEILPR | 72 | 100 |
|  |  |  | -0.0255 | -15 | 208 | 222 | SIEHILILSGDQLYR | 23 | 80.57 |
|  |  |  | -0.0363 | -18 | 232 | 250 | HVDDNADITLSCAPVGES | 24 | 83.04 |
| 44 | [Q0PG36](http://www.agbase.msstate.edu/cgi-bin/getEntry.pl?db_pick=all&database=UniProtKB/TrEMBL&uid=Q0PG36_WHEAT&gb_acc=Q0PG36) | Glucose-1-phosphate adenylyltransferase | 0.1221 | 128 | 380 | 387 | TPFFTSPR | 47 | 99.96 |
|  |  |  | 0.1582 | 125 | 111 | 122 | ATPAVPIGGCYR | 31 | 98.33 |
|  |  |  | 0.1638 | 126 | 401 | 411 | EAIISHGCFLR | 32 | 98.71 |
| 45 | [Q0PG36](http://www.agbase.msstate.edu/cgi-bin/getEntry.pl?db_pick=all&database=UniProtKB/TrEMBL&uid=Q0PG36_WHEAT&gb_acc=Q0PG36) | Glucose-1-phosphate adenylyltransferase | -0.1128 | -119 | 373 | 379 | FEFYDPK | 31 | 98.25 |
|  |  |  | -0.1483 | -118 | 111 | 122 | ATPAVPIGGCYR | 27 | 95.84 |
|  |  |  | -0.1919 | -117 | 321 | 334 | YAELHDFGSEILPR | 61 | 100 |
| **5. Lipid and sterol metabolism:** | | | | | | | | | |
| 30 | gi/167113 | Aldose reductase-related protein | 0.0176 | 9 | 34 | 52 | AGSDTAHSVQTAITEAGYR | 101 | 100 |
| 31 | gi/167113 | Aldose reductase-related protein | 0.0502 | 26 | 33 | 51 | AGSDTAHSVQTAITEAGYR | 116 | 100 |
|  |  |  | 0.0566 | 38 | 140 | 151 | ALEFPDFDKFDR | 71 | 100 |
| 40 | gi/218157 | Cytoplasmic aldolase | -0.0562 | -42 | 238 | 249 | VAPEVIAEYTVR | 45 | 87.36 |
|  |  |  | -0.0616 | -42 | 237 | 249 | KVAPEVIAEYTVR | 35 | 65.68 |
| 145 | gi/66840998 | 5a2 protein | -0.098 | -101 | 77 | 84 | KPFPHGYK | 57 | 99.99 |
|  |  |  | -0.1143 | -107 | 68 | 76 | VAYVANFCK | 42 | 99.86 |
|  |  |  | -0.1233 | -109 | 24 | 32 | YQQWPANPK | 31 | 98.24 |
|  |  |  | -0.1436 | -110 | 46 | 57 | ANIPCLCAGVTK | 58 | 100 |
| **ATP Interconversion** | | | | | | | | | |
| 142 | gi/9652119 | Nucleoside diphosphate kinase | -0.0107 | -11 | 104 | 112 | GDFAVDIGR | 43 | 96.97 |
|  |  |  | -0.0094 | -6 | 88 | 103 | IIGATNPLASEPGTIR | 123 | 100 |
| **Protein synthesis/assembly/degrade** | | | | | | | | | |
| 36 | gi/41052632 | Peptidylprolyl isomerase Cyp2 | -0.0031 | -2 | 6 | 19 | VFFDMTVGGAPAGR | 48 | 95.40 |
|  |  |  | -0.0087 | -6 | 6 | 19 | VFFDMTVGGAPAGR | 50 | 97.08 |
| 54 | gi/154761388 | Cyclophilin | -0.079 | -55 | 6 | 19 | VFFDMTVGGAPAGR | 74 | 99.56 |
|  |  |  | -0.0973 | -59 | 63 | 76 | VIPDFMCQGGDFTR | 15 | 48.36 |
| 55 | gi/154761388 | Cyclophilin | 0.0651 | 46 | 6 | 19 | VFFDMTVGGAPAGR | 61 | 99.99 |
|  |  |  | 0.0471 | 33 | 6 | 19 | VFFDMTVGGAPAGR | 39 | 99.51 |
|  |  |  | 0.0799 | 49 | 63 | 76 | VIPDFMCQGGDFTR | 23 | 82.03 |
| 56 | gi/154761388 | Cyclophilin | -0.0865 | -113 | 32 | 37 | TVENFR | 14 | 49.50 |
|  |  |  | -0.1738 | -122 | 6 | 19 | VFFDMTVGGAPAGR | 81 | 100 |
|  |  |  | -0.1924 | -134 | 6 | 19 | VFFDMTVGGAPAGR | 48 | 99.98 |
| 59 | gi/32352154 | Nascent polypeptide associated complex alpha chain | 0.046 | 27 | 92 | 106 | SKNILFVISKPDVFK | 83 | 99.36 |
| 62 | gi/2492077 | Sequence 5 from patent US 5668007 | -0.0074 | -5 | 15 | 26 | ADANYYVLPANR | 76 | 99.99 |
|  |  |  | -0.0108 | -7 | 1 | 14 | DPPPVHDTDGNELR | 90 | 100 |
| 106 | gi/75279909 | Serpin-Z2B; AltName: Full=TriaeZ2b; | 0.0026 | 3 | 11 | 18 | LSIAHQTR | 22 | 70.88 |
|  |  |  | -0.0537 | -8 | 159 | 171 | DILPAGSIDNTTR | 59 | 100 |
|  |  |  | -0.0201 | -12 | 261 | 274 | LSAEPEFLEQHIPR | 103 | 100 |
|  |  |  | -0.0422 | -20 | 379 | 398 | EDTSGVVLFIGHVVNPLL | 29 | 94.40 |
| 107 | gi/75279909 | Serpin-Z2B; AltName: Full=TriaeZ2b; | 0.0093 | 10 | 11 | 18 | LSIAHQTR | 23 | 82.05 |
|  |  |  | -0.0054 | -5 | 182 | 191 | GAWTDQFDPR | 33 | 98.27 |
|  |  |  | -0.0024 | -2 | 159 | 171 | DILPAGSIDNTTR | 86 | 100 |
|  |  |  | -0.004 | -2 | 261 | 274 | LSAEPEFLEQHIPR | 113 | 100 |
| 128 | gi/12229936 | Proteasome subunit alpha type 7 (20S proteasome alpha subunit D) | -0.1273 | -78 | 97 | 110 | LTVEDPVTVEYITR | 40 | 90.55 |
|  |  |  | -0.0894 | -36 | 7 | 28 | AITVFSPDGHLFQVEYALEAVR | 69 | 99.99 |
| **Storage protein** | | | | | | | | | |
| 67 | gi/171027826 | Triticin | -0.019 | -10 | 211 | 227 | SSQLHSSQNIFSGFDVR | 28 | 88.39 |
|  |  |  | -0.0168 | -8 | 61 | 77 | SQAGLTEYFDEENEQFR | 44 | 99.68 |
| 68 | gi/171027826 | Triticin | -0.0145 | -20 | 92 | 97 | GYLLPR | 23 | 82.62 |
|  |  |  | -0.0421 | -38 | 202 | 210 | EFLFAGNYR | 33 | 98.38 |
|  |  |  | -0.0715 | -44 | 132 | 146 | YGQSQSVQGQSQSQK | 90 | 100 |
|  |  |  | -0.0788 | -41 | 211 | 227 | SSQLHSSQNIFSGFDVR | 100 | 100 |
|  |  |  | -0.0862 | -42 | 61 | 77 | SQAGLTEYFDEENEQFR | 137 | 100 |
|  |  |  | -0.0969 | -46 | 269 | 286 | EPESYPHTQYEEGQSQA | 76 | 100 |
| 69 | gi/7548844 | Triticin precursor | -0.0725 | -57 | 259 | 268 | FLKPVFTQQR | 47 | 95.36 |
|  |  |  | -0.114 | -60 | 211 | 227 | SSQLHSSQNIFSGFDVR | 66 | 99.94 |
|  |  |  | -0.1257 | -61 | 61 | 77 | SQAGLTEYFDEENEQFR | 56 | 99.38 |
| 70 | gi/215398470 | Globulin 3 | -0.0109 | -12 | 535 | 541 | EVQEVFR | 42 | 99.7 |
|  |  |  | -0.016 | -16 | 132 | 139 | RPYVFGPR | 66 | 100 |
|  |  |  | -0.0194 | -16 | 154 | 163 | ALRPFDEVSR | 45 | 99.86 |
|  |  |  | -0.0121 | -9 | 364 | 374 | SFHALAQHDVR | 61 | 100 |
|  |  |  | -0.035 | -26 | 339 | 349 | DTFNLLEQRPK | 29 | 93.40 |
| 71 | gi/215398470 | Globulin 3 | -0.0871 | -117 | 92 | 97 | EEEQGR | 31 | 98.44 |
|  |  |  | -0.1174 | -122 | 48 | 54 | CQQDRPR | 14 | 20.08 |
|  |  |  | -0.1192 | -121 | 66 | 73 | DDQQQHGR | 38 | 99.67 |
|  |  |  | -0.1212 | -120 | 74 | 81 | HEQEEQGR | 22 | 87.48 |
|  |  |  | -0.1847 | -131 | 86 | 97 | HGEGEREEEQGR | 36 | 99.46 |
| 72 | gi/89143122 | Putative avenin-like b precursor | -0.0108 | -13 | 249 | 255 | MSLQALR | 20 | 59.79 |
|  |  |  | -0.0262 | -21 | 203 | 212 | QLSQIPEQFR | 58 | 99.99 |
|  |  |  | -0.0283 | -20 | 213 | 224 | CQAIHNVAEAIR | 88 | 100 |
| 73 | gi/145321072 | Avenin-like protein | -0.1255 | -101 | 202 | 211 | QLSQIPEQFR | 62 | 100 |
|  |  |  | -0.1447 | -105 | 212 | 223 | CQAIHNVAEAIR | 35 | 99.06 |
| 74 | gi/209971847 | Gamma-gliadin | -0.1565 | -132 | 235 | 247 | APFSSVVAGIGGQ | 70 | 100 |
| 75 | gi/133741924 | Gamma gliadin | 0.1169 | 60 | 60 | 76 | DALLQQCSPVADMSFLR | 93 | 100 |
|  |  |  | 0.1038 | 53 | 60 | 76 | DALLQQCSPVADMSFLR | 21 | 75.68 |
| 76 | gi/133741924 | Gamma gliadin | -0.1408 | -72 | 60 | 76 | DALLQQCSPVADMSFLR | 52 | 99.99 |
|  |  |  | -0.1559 | -79 | 60 | 76 | DALLQQCSPVADMSFLR | 21 | 89.22 |
| 77 | gi/209971907 | Gamma-gliadin | 0.1968 | 83 | 184 | 202 | SDCQVMQQQCCQQLAQI | 55 | 99.99 |
|  |  |  | 0.1949 | 81 | 184 | 202 | SDCQVMQQQCCQQLAQI | 41 | 99.80 |
| 78 | gi/164470668 | LMW-s glutenin subunit 0359D24-S | 0.0722 | 84 | 332 | 338 | VNVPLYR | 33 | 97.89 |
|  |  |  | 0.147 | 71 | 199 | 216 | VFLQQQCSPVAMPQSLA | 24 | 83.69 |
| 79 | gi/47607146 | S-type low molecular weight glutenin L4-55 | 0.0637 | 74 | 224 | 230 | VNVPLYR | 34 | 98.57 |
|  |  |  | 0.1323 | 64 | 91 | 108 | VFLQQQCSPVAMPQSLA | 56 | 99.99 |
| 80 | gi/215398468 | Globulin 3C | 0.1559 | 154 | 74 | 81 | HEQEEQGR | 23 | 83.10 |
|  |  |  | 0.1957 | 154 | 37 | 47 | GGHSLQQCVQR | 57 | 99.99 |
| 81 | gi/421978 | Globulin Beg1 precursor | 0.0543 | 60 | 581 | 587 | EVQEVFR | 32 | 94.46 |
|  |  |  | 0.0582 | 59 | 181 | 188 | RPYVFGPR | 31 | 12.61 |
| 82 | gi/167004 | Embryo globulin | -0.039 | -43 | 581 | 587 | EVQEVFR | 37 | 98.66 |
| 84 | gi/170696 | Storage protein | 0.0192 | 19 | 181 | 188 | RPYVFGPR | 52 | 98.08 |
| **Nitrogen metabolism** | | | | | | | | | |
| 47 | gi/164471780 | Aspartate aminotransferase | 0.012 | 14 | 94 | 101 | VGGEFLAR | 70 | 99.87 |
|  |  |  | 0.0056 | 4 | 66 | 79 | LIFGADSPAIQENR | 15 | 30.45 |
| 48 | gi/164471780 | Aspartate aminotransferase | -0.1605 | -110 | 22 | 34 | TEEGKPLVLNVVR | 32 | 88.98 |
|  |  |  | -0.1529 | -100 | 66 | 79 | LIFGADSPAIQENR | 63 | 98.76 |
| 49 | gi/584706 | Aspartate aminotransferase, cytoplasmic | -0.0476 | -56 | 115 | 122 | VGGEFLAR | 33 | 27.99 |
|  |  |  | -0.0828 | -57 | 43 | 55 | TEEGKPLVLNVVR | 43 | 93.25 |
|  |  |  | -0.0864 | -56 | 87 | 100 | LIFGADSPAIQENR | 61 | 99.89 |
| 51 | gi/14018051 | Putative alanine aminotransferase | -0.0286 | -37 | 464 | 470 | IPAIISR | 34 | 40.85 |
|  |  |  | -0.0257 | -25 | 421 | 429 | APDAFYALR | 38 | 78.72 |
|  |  |  | -0.0403 | -27 | 311 | 324 | GGYMEITGFSAPVR | 35 | 55.95 |
| 52 | gi/56315117 | Predicted serine-pyruvate aminotransferase | 0.025 | 18 | 4 | 20 | HPDPDGLLEYSVVYTDR | 113 | 99.99 |
| 53 | gi/6006863 | Putative methionine synthase | -0.0437 | -45 | 393 | 401 | VTNESVQK | 69 | 99.34 |
| **Transcription/translation** | | | | | | | | | |
| 60 | gi/479830 | Translation elongation factor eEF-1 beta' chain | 0.0084 | 6 | 2 | 15 | AVTFSDLHTADGLK | 64 | 99.96 |
| 85 | gi/18419557 | Transposase | -0.055 | -74 | 94 | 99 | VGFIFR | 17 | 34.42 |
|  |  |  | -0.0673 | -74 | 76 | 82 | QVDYLIR | 18 | 48.15 |
|  |  |  | -0.0784 | -81 | 147 | 154 | IIGFDNMR | 18 | 38.51 |
|  |  |  | -0.0812 | -80 | 139 | 146 | EYPDAYVR | 27 | 93.34 |
| 86 | gi/30793446 | 27K protein | 0.0058 | 5 | 133 | 142 | GHNLSLEYGR | 71 | 99.98 |
| 87 | gi/30793446 | 27K protein | 0.0192 | 17 | 133 | 142 | GHNLSLEYGR | 69 | 99.96 |
| 88 | gi/30793446 | 27K protein | -0.0437 | -45 | 28 | 35 | FVANHLFK | 27 | 94.29 |
|  |  |  | -0.0412 | -38 | 181 | 189 | GYPLLEACR | 21 | 78.8 |
|  |  |  | -0.0444 | -39 | 133 | 142 | GHNLSLEYGR | 55 | 99.99 |
|  |  |  | -0.0975 | -55 | 90 | 104 | VHLGFIYCVSDLVLK | 68 | 100 |
| 89 | gi/46394372 | TPA: TPA_inf: WRKY transcription factor 59 | 0.1371 | 146 | 174 | 182 | CSTEGCNVK | 65 | 95.99 |
|  |  |  | -0.0912 | -68 | 132 | 142 | TMTDKIAFRTR | 58 | 90.06 |
| 90 | gi/30793446 | 27K protein | -0.0561 | -49 | 133 | 142 | GHNLSLEYGR | 60 | 99.69 |
| 91 | gi/27735373 | Replication factor C like protein | 0.0317 | 33 | 303 | 311 | FVFGGGKSR | 64 | 96.12 |
| 93 | [Q03033](http://www.agbase.msstate.edu/cgi-bin/getEntry.pl?db_pick=all&database=UniProtKB/Swiss-Prot&uid=EF1A_WHEAT&gb_acc=Q03033) | Elongation factor 1-alpha | -0.0015 | -1 | 85 | 96 | YYCTVIDAPGHR | 67 | 100 |
|  |  |  | 0.0097 | 4 | 255 | 278 | VETGVIKPGMVVTFGPTG | 24 | 83.24 |
| **Signal transduction** | | | | | | | | | |
| 64 | gi/134290443 | Pm3b-like disease resistance protein 15Q1 | -0.0912 | -68 | 1090 | 1098 | VFQSMVSLR | 41 | 78.80 |
|  |  |  | -0.0561 | -49 | 1288 | 1298 | NCAGMLGGPLR | 55 | 87.95 |
| 65 | gi/18145 | Putative protein has homology to G protein beta subunit | 0.0462 | 35 | 108 | 119 | DVLSVAFSVDNR | 60 | 99.99 |
| 66 | gi/50932677 | Putative guanine nucleotide-binding protein beta subunit | 0.1289 | 128 | 144 | 152 | LWNTLGECK | 65 | 100 |
|  |  |  | 0.1446 | 130 | 174 | 190 | FSPNPMAPTIVSGSWDR | 56 | 99.41 |
| **Stress/defense** | | | | | | | | | |
| 57 | gi/640015 | CMx | -0.1493 | -177 | 27 | 33 | EQCVPGR | 24 | 96.78 |
|  |  |  | -0.1692 | -176 | 112 | 120 | EFIAGIVGR | 79 | 100 |
| 58 | gi/75107149 | RecName: Full=Chymotrypsin inhibitor WCI Full=Chloroform/methanol-soluble protein WCI | -0.0685 | -61 | 47 | 56 | ELAAISSNCR | 51 | 99.98 |
|  |  |  | -0.1204 | -61 | 67 | 86 | AFPPSQSQGGGPPQPPL | 41 | 99.78 |
|  |  |  | -0.1579 | -65 | 98 | 119 | TLALPGQCNLPTIHGGPY | 72 | 100 |
| 94 | gi/38098487 | Alpha amylase inhibitor protein | 0.1289 | 128 | 37 | 44 | TNLLPHCR | 35 | 99.05 |
|  |  |  | 0.1446 | 130 | 133 | 140 | EMQWDFVR | 27 | 94.66 |
| 95 | gi/66841026 | Alpha-amylase inhibitor 0.19 | -0.1434 | -91 | 21 | 34 | LQCNGSQVPEAVLR | 36 | 99.41 |
|  |  |  | -0.1495 | -93 | 62 | 77 | EHGAQEGQAGTGAFPR | 59 | 100 |
| 96 | gi/66841026 | Alpha-amylase inhibitor 0.19 | -0.0824 | -71 | 85 | 95 | LTAASITAVCR | 54 | 99.99 |
|  |  |  | -0.1347 | -86 | 21 | 34 | LQCNGSQVPEAVLR | 82 | 100 |
|  |  |  | -0.1426 | -88 | 62 | 77 | EHGAQEGQAGTGAFPR | 135 | 100 |
|  |  |  | -0.1512 | -91 | 96 | 111 | LPIVVDASGDGAYVCK | 129 | 100 |
|  |  |  | -0.1549 | -83 | 35 | 48 | DCCQQLAHISEWCR | 95 | 100 |
| 97 | gi/123956 | Alpha-amylase/trypsin inhibitor CM2; | -0.0214 | -26 | 107 | 113 | DLPGCPR | 29 | 96.66 |
|  |  |  | -0.0455 | -36 | 77 | 86 | ELYDASQHCR | 66 | 100 |
| 98 | gi/221855644 | Alpha-amylase inhibitor CM16 subunit | -0.1615 | -158 | 107 | 114 | EVQMDFVR | 27 | 97.43 |
|  |  |  | -0.1819 | -156 | 45 | 53 | DYVEQQACR | 33 | 99.33 |
| 99 | gi/221855644 | Alpha-amylase inhibitor CM16 subunit | -0.1428 | -140 | 107 | 114 | EVQMDFVR | 59 | 100 |
|  |  |  | -0.1682 | -144 | 45 | 53 | DYVEQQACR | 38 | 99.83 |
| 100 | gi/225042 | Alpha amylase inhibitor | -0.0317 | -23 | 15 | 26 | ADANYYVLPANR | 20 | 30.61 |
|  |  |  | -0.0461 | -30 | 1 | 14 | DPPPVHDTDGNELR | 59 | 95.60 |
| 101 | gi/123955 | Alpha-amylase/trypsin inhibitor CM1; | -0.0415 | -32 | 77 | 86 | ELYDASQHCR | 31 | 98.49 |
|  |  |  | -0.0989 | -37 | 46 | 71 | EYVAQQTCGISISGSAVS | 19 | 75.68 |
| 102 | gi/54778507 | 0.19 dimeric alpha-amylase inhibitor | -89 | 101 | 116 | 114 | LPIVIDASGDGAYVCK | 114 | 100 |
|  |  |  | -82 | 67 | 84 | 124 | EHGVQEGQAGTGAFPSC | 124 | 100 |
| 104 | gi/408873 | Puroindoline=basic cystine-rich protein | -0.0143 | -16 | 33 | 39 | DFPVTWR | 24 | 87.22 |
|  |  |  | -0.0123 | -11 | 59 | 67 | LGQMPPQCR | 17 | 26.28 |
|  |  |  | -0.0223 | -15 | 46 | 58 | GGCQELLGECCSR | 31 | 97.47 |
|  |  |  | -0.0364 | -17 | 68 | 87 | CNIIQGSIQGDLGGIFGFQ | 36 | 99.14 |
| 105 | gi/408873 | Puroindoline=basic cystine-rich protein | -0.0143 | -16 | 33 | 39 | DFPVTWR | 24 | 87.22 |
|  |  |  | -0.0123 | -11 | 59 | 67 | LGQMPPQCR | 17 | 26.28 |
|  |  |  | -0.0223 | -15 | 46 | 58 | GGCQELLGECCSR | 31 | 97.47 |
|  |  |  | -0.0364 | -17 | 68 | 87 | CNIIQGSIQGDLGGIFGFQ | 36 | 99.14 |
| 110 | gi/51247633 | Chain A, Crystal Structure Of Family 11 Xylanase In Complex With Inhibitor (Xip-I) | 0.0065 | 5 | 235 | 246 | NVYYGVAPVAQK | 32 | 96.73 |
|  |  |  | 0.0121 | 6 | 47 | 64 | YHLDLSGHDLSSVGADIK | 44 | 99.80 |
| 111 | gi/51247633 | Chain A, Crystal Structure Of Family 11 Xylanase In Complex With Inhibitor (Xip-I) | -0.006 | -6 | 175 | 183 | ALATGIFER | 20 | 66.83 |
|  |  |  | -0.0256 | -20 | 235 | 246 | NVYYGVAPVAQK | 22 | 79.45 |
|  |  |  | -0.0266 | -14 | 47 | 64 | YHLDLSGHDLSSVGADIK | 107 | 100 |
| 112 | gi/62465514 | Class II chitinase | 0.0023 | 1 | 138 | 153 | GPIQLSHNYNYGPAGR | 66 | 100 |
|  |  |  | -0.0278 | -12 | 154 | 175 | AIGVDLLSNPDLVATDPT | 28 | 95.11 |
| 113 | gi/62465514 | Class II chitinase | -0.0095 | -5 | 138 | 153 | GPIQLSHNYNYGPAGR | 54 | 99.96 |
| 114 | gi/62465514 | Class II chitinase | 0.013 | 7 | 138 | 153 | GPIQLSHNYNYGPAGR | 59 | 99.99 |
|  |  |  | -0.024 | -9 | 245 | 266 | YCDILGVGYGDNLDCYN | 59 | 100 |
| 115 | gi/62465514 | Class II chitinase | -0.1579 | -65 | 98 | 119 | TLALPGQCNLPTIHGGPY | 72 | 100 |
|  |  |  | 0.0095 | 4 | 211 | 234 | VPGFGVITNIINGGIECGH | 28 | 95.27 |
|  |  |  | 0.0085 | 3 | 245 | 266 | YCDILGVGYGDNLDCYN | 32 | 97.92 |
| 116 | gi/62465514 | Class II chitinase | -0.0478 | -27 | 138 | 153 | GPIQLSHNYNYGPAGR | 77 | 100 |
|  |  |  | -0.0776 | -34 | 154 | 175 | AIGVDLLSNPDLVATDPT | 94 | 100 |
|  |  |  | -0.073 | -29 | 211 | 234 | VPGFGVITNIINGGIECGH | 20 | 73.07 |
|  |  |  | -0.0799 | -31 | 245 | 266 | YCDILGVGYGDNLDCYN | 15 | 6.41 |
| 117 | gi/25989705 | LEA1 protein | -0.0485 | -25 | 169 | 188 | TGSVLQQAGETVVNAVV | 76 | 100 |
|  |  |  | -0.0425 | -20 | 167 | 188 | DKTGSVLQQAGETVVNA | 28 | 89.14 |
| 119 | gi/134034615 | Monomeric alpha-amylase inhibitor | -0.102 | -123 | 77 | 83 | EVLPGCR | 15 | 20.35 |
|  |  |  | -0.1369 | -130 | 65 | 73 | SVYQELGVR | 31 | 97.96 |
| 120 | gi/134034615 | Monomeric alpha-amylase inhibitor | -0.0914 | -110 | 77 | 83 | EVLPGCR | 24 | 90.35 |
|  |  |  | -0.1246 | -119 | 65 | 73 | SVYQELGVR | 23 | 87.85 |
| 121 | gi/22001285 | Peroxidase 1 | -0.1 | -100 | 62 | 71 | DIGLAAGLLR | 43 | 95.16 |
|  |  |  | -0.1024 | -102 | 34 | 41 | GLSFDFYR | 22 | 45.56 |
|  |  |  | -0.1657 | -118 | 298 | 309 | DFFEQFGVSMGK | 29 | 89.17 |
| 122 | gi/22001285 | Peroxidase 1 | -0.0117 | -13 | 53 | 60 | GFVQDAVR | 19 | 44.40 |
|  |  |  | -0.017 | -17 | 62 | 71 | DIGLAAGLLR | 26 | 89.18 |
|  |  |  | -0.0193 | -19 | 34 | 41 | GLSFDFYR | 38 | 99.34 |
|  |  |  | -0.0188 | -15 | 142 | 153 | DSVVVSGGPDYR | 28 | 93.18 |
| 123 | gi/22001285 | Peroxidase 1 | -0.0995 | -100 | 62 | 71 | DIGLAAGLLR | 31 | 97.78 |
|  |  |  | -0.0999 | -99 | 34 | 41 | GLSFDFYR | 25 | 89.91 |
|  |  |  | -0.124 | -99 | 142 | 153 | DSVVVSGGPDYR | 25 | 90.09 |
|  |  |  | -0.1578 | -113 | 298 | 309 | DFFEQFGVSMGK | 25 | 89.91 |
|  |  |  | -0.1604 | -114 | 298 | 309 | DFFEQFGVSMGK | 33 | 98.60 |
|  |  |  | -0.152 | -102 | 127 | 141 | GAVVSCADILALAAR | 22 | 82.66 |
| 124 | gi/75246527 | Translationally-controlled tumor protein | -0.0421 | -40 | 67 | 75 | VVDIVDTFR | 56 | 99.99 |
|  |  |  | -0.0684 | -43 | 8 | 21 | LSGDELLSDSFPYR | 80 | 100 |
|  |  |  | -0.0832 | -48 | 149 | 164 | EGAADPTFLYFAHGLK | 60 | 100 |
| 125 | gi/20257409 | Thaumatin-like protein | 0.0674 | 64 | 70 | 79 | TGCTFDGSGR | 30 | 96.22 |
|  |  |  | 0.0725 | 64 | 168 | 176 | FGGDTYCCR | 37 | 99.22 |
|  |  |  | 0.102 | 61 | 177 | 190 | GQFEHNCPPTNYSK | 50 | 99.96 |
|  |  |  | 0.1191 | 58 | 95 | 114 | VSGQQPTTLAEYTLGQG | 75 | 100 |
|  |  |  | 0.1897 | 67 | 115 | 139 | DFFDLSVIDGFNVPMNFE | 63 | 100 |
|  |  |  | 0.188 | 66 | 115 | 139 | DFFDLSVIDGFNVPMNFE | 48 | 99.94 |
| 126 | gi/20257409 | Thaumatin-like protein | -0.0284 | -27 | 70 | 79 | TGCTFDGSGR | 79 | 100 |
|  |  |  | -0.0251 | -22 | 168 | 176 | FGGDTYCCR | 56 | 99.70 |
| 127 | gi/226897529 | Superoxide dismutase | -0.01 | -8 | 115 | 127 | AVVVHGDADDLGK | 64 | 100 |
|  |  |  | -0.029 | -12 | 16 | 38 | GTIFFTQEGEGPTTVTGS | 17 | 34.55 |
| **Photosynthesis** | | | | | | | | | |
| 132 | gi/11990897 | Ribulose-1,5-bisphosphate carboxylase/oxygenase small subunit | -0.055 | -74 | 94 | 99 | VGFIFR | 17 | 34.42 |
|  |  |  | -0.0673 | -74 | 76 | 82 | QVDYLIR | 18 | 48.15 |
|  |  |  | -0.0784 | -81 | 147 | 154 | IIGFDNMR | 18 | 38.51 |
|  |  |  | -0.0812 | -80 | 139 | 146 | EYPDAYVR | 27 | 93.34 |
|  |  |  | -0.1114 | -96 | 85 | 93 | WVPCLEFSK | 40 | 99.66 |
|  |  |  | -0.1165 | -85 | 100 | 111 | EHNASPGYYDGR | 65 | 100 |
|  |  |  | -0.183 | -95 | 59 | 75 | FETLSYLPPLSTEALLK | 23 | 81.30 |
| 140 | gi/34393258 | Putative Oxygen-evolving enhancer protein 3-1, chloroplast precursor | 0.0586 | 43 | 175 | 187 | LFATIDGLDHAAK | 59 | 99.77 |
| **Others** | | | | | | | | | |
| 143 | gi/357152329 | Probable beta-D-xylosidase 7-like | 0.0088 | 9 | 405 | 413 | NDAGILPLR | 14 | 26.45 |
|  |  |  | 0.0105 | 9 | 569 | 579 | VLFGDHNPSGR | 29 | 95.21 |
| 146 | gi/2454602 | Barperm1 | -0.0374 | -35 | 50 | 59 | TGCTFDGSGR | 44 | 95.10 |
|  |  |  | -0.0315 | -28 | 148 | 156 | FGGDTYCCR | 34 | 59.24 |
|  |  |  | -0.0357 | -25 | 62 | 74 | CITGDCGGALACR | 59 | 99.86 |
|  |  |  | -0.0455 | -27 | 157 | 170 | GQFEHNCPPTNYSK | 70 | 99.99 |
| 147 | gi/115458852 | Os04g0465600 | -0.0633 | -35 | 81 | 96 | VVSYSVVDGELVSFYK | 82 | 100 |
