# Supplementary material for: Proteome characterization of developing grains in bread wheat cultivars (Triticum aestivum L.)
Source: BMC Plant Biol. 2012 Aug 19;12:147. doi: 10.1186/1471-2229-12-147 (PMC3480910; doi:10.1186/1471-2229-12-147)
Supplement: Additional file 2 — The peptide sequences of phosphorylated proteins during wheat grain development identified by MS/MS. [file 1471-2229-12-147-S2.doc]

**Appendix Table 2 The peptide sequences of phosphorylated proteins during wheat grain development identified by MS/MS**

| **Spot**  **No.** | **Accession**  **No.** | **Protein name** | **±da** | **±ppm** | **Start**  **Sequence** | **End**  **Sequence** | **Peptide Sequence** | **Ion Score** | **Ion Score**  **C. I%** |
| --- | --- | --- | --- | --- | --- | --- | --- | --- | --- |
| 67^*^ | gi/171027826 | Triticin | -0.019 | -10 | 211 | 227 | SSQLHSSQNIFSGFDVR | 28 | 88.39 |
|  |  |  | -0.0168 | -8 | 61 | 77 | SQAGLTEYFDEENEQFR | 44 | 99.68 |
| 70^*^ | gi/215398470 | Globulin 3 | -0.0109 | -12 | 535 | 541 | EVQEVFR | 42 | 99.70 |
|  |  |  | -0.016 | -16 | 132 | 139 | RPYVFGPR | 66 | 100 |
|  |  |  | -0.0194 | -16 | 154 | 163 | ALRPFDEVSR | 45 | 99.86 |
|  |  |  | -0.0121 | -9 | 364 | 374 | SFHALAQHDVR | 61 | 100 |
|  |  |  | -0.035 | -26 | 339 | 349 | DTFNLLEQRPK | 29 | 93.40 |
|  |  |  | -0.019 | -10 | 489 | 504 | GSSNLQVVCFEINAER | 120 | 100 |
| 94^*^ | gi/38098487 | Alpha amylase inhibitor protein | 0.0573 | 51 | 133 | 140 | EMQWDFVR | 14 | 43.78 |
|  |  |  | 0.094 | 55 | 101 | 115 | YFIALPVPSQPVDPR | 65 | 100 |
|  |  |  | 0.0952 | 55 | 116 | 132 | SGNVGESGLIDLPGCPR | 73 | 100 |
| 96^*^ | gi/54778501 | 0.19 dimeric alpha-amylase inhibitor | 0.0846 | 54 | 26 | 39 | LQCNGSQVPEAVLR | 58 | 100 |
|  |  |  | 0.0841 | 52 | 67 | 82 | EHGAQEGQAGTGAFPR | 101 | 100 |
|  |  |  | 0.0749 | 45 | 101 | 116 | LPIVVDASGDGAYVCK | 134 | 100 |
|  |  |  | 0.099 | 53 | 40 | 53 | DCCQQLAHISEWCR | 67 | 100 |
| 100^*^ | gi/225042 | Alpha amylase inhibitor | 0.0859 | 63 | 15 | 26 | ADANYYVLPANR | 81 | 100 |
|  |  |  | 0.0892 | 59 | 42 | 54 | CPLFVSQEADGQR | 89 | 100 |
|  |  |  | 0.0828 | 53 | 1 | 14 | DPPPVHDTDGNELR | 69 | 100 |
|  |  |  | 0.0972 | 56 | 140 | 154 | LMACGDSCQDLGVFR | 20 | 86.64 |
| 102^*^ | gi/54778507 | 0.19 dimeric alpha-amylase inhibitor | 0.0907 | 58 | 26 | 39 | LQCNGSQVPEAVLR | 79 | 100 |
|  |  |  | 0.0825 | 49 | 101 | 116 | LPIVIDASGDGAYVCK | 124 | 100 |
|  |  |  | 0.114 | 61 | 40 | 53 | ECCQQLADISEWCR | 56 | 100 |
|  |  |  | 0.1072 | 57 | 67 | 84 | EHGVQEGQAGTGAFPSCR | 113 | 100 |
| 112^*^ | gi/62465514 | Class II chitinase | 0.0023 | 1 | 138 | 153 | GPIQLSHNYNYGPAGR | 66 | 100 |
|  |  |  | -0.0278 | -12 | 154 | 175 | AIGVDLLSNPDLVATDPTK | 28 | 95.11 |
| 113^*^ | gi/62465514 | Class II chitinase | 0.0784 | 31 | 211 | 234 | VPGFGVITNIINGGIECGHR | 69 | 99.38 |
| 114^*^ | gi/62465514 | Class II chitinase | 0.013 | 7 | 138 | 153 | GPIQLSHNYNYGPAGR | 59 | 100 |
|  |  |  | -0.024 | -9 | 245 | 266 | YCDILGVGYGDNLDCYNK | 59 | 100 |
| 122^*^ | gi/22001285 | Peroxidase 1 | 0.1501 | 150 | 62 | 71 | DIGLAAGLLR | 46 | 99.95 |
|  |  |  | 0.1514 | 151 | 34 | 41 | GLSFDFYR | 44 | 99.91 |
|  |  |  | 0.1903 | 152 | 142 | 153 | DSVVVSGGPDYR | 48 | 99.96 |
|  |  |  | 0.084 | 32 | 245 | 266 | YCDILGVGYGDNLDCYN | 39 | 99.46 |
| 123^*^ | gi/22001285 | Peroxidase 1 | 0.1454 | 146 | 62 | 71 | DIGLAAGLLR | 45 | 99.92 |
|  |  |  | 0.1457 | 145 | 34 | 41 | GLSFDFYR | 44 | 99.89 |
|  |  |  | 0.1846 | 148 | 142 | 153 | DSVVVSGGPDYR | 74 | 100 |
|  |  |  | 0.1907 | 135 | 298 | 309 | DFFEQFGVSMGK | 43 | 99.89 |
| A^#^ | gi/4558484 | Heat shock protein 101 | 0.0256 | 17 | 484 | 495 | EELQFTLQEAER | 26 | 92.01 |
|  |  |  | 0.0232 | 13 | 620 | 634 | ALAEQLFDDENLLVR | 65 | 100 |
|  |  |  | 0.017 | 10 | 277 | 292 | VILFIDEIHLVLGAGR | 46 | 99.92 |
|  |  |  | 0.0256 | 14 | 407 | 421 | VQLDSQPEEIDNLER | 73 | 100 |
|  |  |  | 0.0215 | 11 | 761 | 776 | LDEIVVFDPLSHEQLR | 44 | 99.86 |
|  |  |  | 0.0242 | 10 | 649 | 670 | LIGAPPGYVGHEEGGQLR | 33 | 98.46 |
| B^#^ | gi/4204859 | Heat shock protein 80 | 0.028 | 26 | 320 | 328 | APFDLFDTR | 46 | 99.90 |
|  |  |  | 0.0314 | 26 | 58 | 67 | LDAQPELFIR | 62 | 100 |
|  |  |  | 0.0184 | 15 | 319 | 328 | RAPFDLFDTR | 26 | 88.17 |
|  |  |  | 0.0137 | 10 | 301 | 311 | HFSVEGQLEFK | 85 | 100 |
|  |  |  | 0.0277 | 18 | 360 | 373 | GIVDSEDLPLNISR | 53 | 99.98 |
| C^#^ | gi/18146829 | Chitinase 3 | 0.0918 | 35 | 298 | 319 | YCDLLGVSYGDNLDCYN | 63 | 100 |
| D^#^ | gi/34925030 | RecName: Full=Wheatwin-1; | 0.0793 | 47 | 87 | 102 | CLQVTNPATGAQITAR | 74 | 100 |
|  |  |  | 0.0979 | 43 | 103 | 122 | IVDQCANGGLDLDWDTVR | 111 | 100 |
|  |  |  | 0.148 | 55 | 123 | 145 | IDTNGIGYQQGHLNVNYQR | 39 | 99.83 |
|  |  |  | 0.1734 | 61 | 123 | 146 | IDTNGIGYQQGHLNVNYQR | 51 | 99.99 |
| E^#^ | gi/34925032 | RecName: Full=Wheatwin-2; | 0.073 | 56 | 92 | 104 | VTNPATGAQITAR | 65 | 100 |
|  |  |  | 0.0984 | 43 | 105 | 124 | IVDQCANGGLDLDWDTVK | 98 | 100 |
|  |  |  | 0.1534 | 57 | 125 | 147 | IDTNGIGYQQGHLNVNYQR | 24 | 94.03 |
|  |  |  | 0.148 | 52 | 125 | 148 | IDTNGIGYQQGHLNVNYQR | 39 | 99.85 |

*the numbers of spots correspond to spot No. shown in Fig. 3.

^#^the newly identified protein spots of phosphorylation.
