# Supplementary material for: Proteome characterization of developing grains in bread wheat cultivars (Triticum aestivum L.)
Source: BMC Plant Biol. 2012 Aug 19;12:147. doi: 10.1186/1471-2229-12-147 (PMC3480910; doi:10.1186/1471-2229-12-147)
Supplement: Additional file 3 — Primer sequences used for quantitative real-time RT-PCR (qRT-PCR). [file 1471-2229-12-147-S3.doc]

**Table 5 Primer sequences used for quantitative real-time RT-PCR (qRT-PCR)**

| Spot no^a)^ | Protein name | Forward primer (5’-3’) | Reverse primer (5’-3’) |
| --- | --- | --- | --- |
| 18,19 | Phosphoglucomutase | TCTCCAAGCATCAGGGTATCC | CTCAATGTAAATACGGATAGTAGCAC |
| 20 | Fructose-bisphosphate aldolase | TGCTGGAACCAACGGTGA | AGCAGATGATGGCATAGCG |
| 22,23 | Cytosolic malate dehydrogenase | AACCCAGCAAACACCAATCC | CAGAAATCTGACCGAGTGCC |
| 41, 44,45 | Glucose-1-phosphate adenylyltransferase | TGACGAGACCAGGGAAGAGTA | AAACTTCCCACTGAGGATAGCA |
| 54,55,56 | Cyclophilin | ACACCAACGGCTCCCAGT | AGGCGAACGGATCTAGAGC |
| 94 | Alpha amylase inhibitor protein | ACAAACTTGTGGCACCTTCAC | AGCTCCTGGCAGCAATACA |
| 119,120 | Monomeric alpha-amylase inhibitor | TCGCCGACATCAACAACG | ACGCCGAGTTCCTGATACA |
| 125,126 | Thaumatin-like protein | CTCCACCTCATCGCCCTC | CCACACCGTGTAGGAGCAAC |
| 127 | Superoxide dismutase | CAAGGGTGGACATGAGCTGA | AAAGGTTCCCATATCTGTACGAC |
| 143 | Probable beta-D-xylosidase 7-like | TTGGTGGCATTGGAACTGT | GATCCTTCACGGCAACATTC |

^a^Protein spots as showed in Table 2.
